# Elucidation of the mechanism of Jinmaitong against Diabetic peripheral neuropathy based on a combined strategy of network pharmacology and molecular biology

**DOI:** 10.1186/s13020-025-01300-0

**Published:** 2026-02-02

**Authors:** Ziman Yu, Bingjia Zhao, Wei Song, Hangqi Liu, Yanfei Che, Dongshan Qin, Xiaochun Liang, Dan Yang

**Affiliations:** 1 Department of Traditional Chinese Medicine, Peking Union Medical College Hospital, Chinese Academy of Medical Sciences and Peking Union Medical College, Beijing, 100730 China; 2 Institute of Clinical Medicine, National Infrastructures for Translational Medicine, Peking Union Medical College Hospital, Chinese Academy of Medical Sciences and Peking Union Medical College, Beijing, 100730 China; 3 Department of Pathology, Peking Union Medical College Hospital, Chinese Academy of Medical Sciences and Peking Union Medical College, Beijing, 100730 China

**Keywords:** Diabetic peripheral neuropathy, Jinmaitong, JAK2/STAT3 signaling, Macrophage polarization, Neuroinflammation

## Abstract

**Background:**

Diabetic peripheral neuropathy (DPN) is a common complication of type 2 diabetes mellitus (T2DM) with limited treatment options. The traditional Chinese medicine Jinmaitong (JMT) has demonstrated efficacy in treating DPN in both clinical and animal studies. It is worth noting that macrophage polarization appears to play a significant role in the onset and progression of DPN. However, whether the specific mechanism by which JMT exerts its neuroprotective effects is related to macrophage polarization still requires further in-depth investigation.

**Methods:**

T2DM model was established using Sprague–Dawley (SD) rats induced by a high-fat diet for six weeks combined with streptozotocin (STZ) injection. After modeling and drug administration, the DPN status was assessed using the von Frey test to test mechanical threshold, the hot plate test and tail flick test to evaluate thermal response latency, and the bioelectric amplifier to measure motor nerve conduction velocity.

In the first batch of *in-vivo* experiments (Batch 1), after establishing the type 2 diabetes model, we conducted herbal formula JMT administered daily via oral gavage for another four weeks, eight weeks or twelve weeks, with each study comprising four groups: control group (CON), DPN group (DPN), low-dose JMT (7.6 mg/kg) treated group (DPN + JMT), and high-dose JMT (15.2 mg/kg) treated group (DPN + JMTH). The pharmacological effects of JMT on neurological function, neuropathology, and the levels of M1 and M2 macrophage cytokine markers were evaluated in serum and sciatic nerve, respectively. After chemical profiling of JMT by liquid chromatography coupled with high-resolution mass spectrometry, network pharmacology analysis was subsequently employed to predict the potential signaling pathways that JMT targeted in treating DPN. We further explored JMT’s neuroprotective effect in a second batch of *in-vivo* experiments. To do this, we co-administered the JAK2/STAT3 inhibitor AG490 along with macrophage polarizing agents: LPS and interleukin-4 (IL-4). The changes of M1 and M2 macrophages in bone marrow was investigated by cytometry, while the macrophages in sciatic nerves were observed by immunofluorescence. Myelin morphology was observed with Luxel fast blue staining and transmission electron microscopy. Immunofluorescence was performed to evaluate nerve injury and regeneration, with S100 and neurofilament 160 (NF160) used to label Schwann cells and axons respectively in the sciatic nerve. The protein expressions of JAK2/STAT3 signaling in sciatic nerves were examined by Western blot.

**Results:**

JMT significantly improved neurological function and pathological damage in type 2 DPN rats. Eight weeks after diabetes induction, DPN rats showed a significant increase in pro-inflammatory cytokines and a concurrent decrease in anti-inflammatory cytokines. JMT administration effectively restored the imbalance. Furthermore, JMT reduced the proportion of M1 macrophages while increasing that of M2 macrophages. JMT promoted the polarization of macrophages from the M1 to the M2 phenotype in both bone marrow-derived macrophages and those infiltrating the sciatic nerve, which was mediated through the suppression of abnormal activation of the JAK2/STAT3 signaling pathway.

**Conclusions:**

JMT promotes the polarization of macrophages from the M1 to M2 phenotype and alleviates neuroinflammation in T2DM rats with DPN, which is associated with inhibition of the JAK2/STAT3 signaling pathway. These findings highlight the neuroprotective potential of JMT through immunomodulatory mechanisms.

**Graphical Abstract:**

**Figure:**
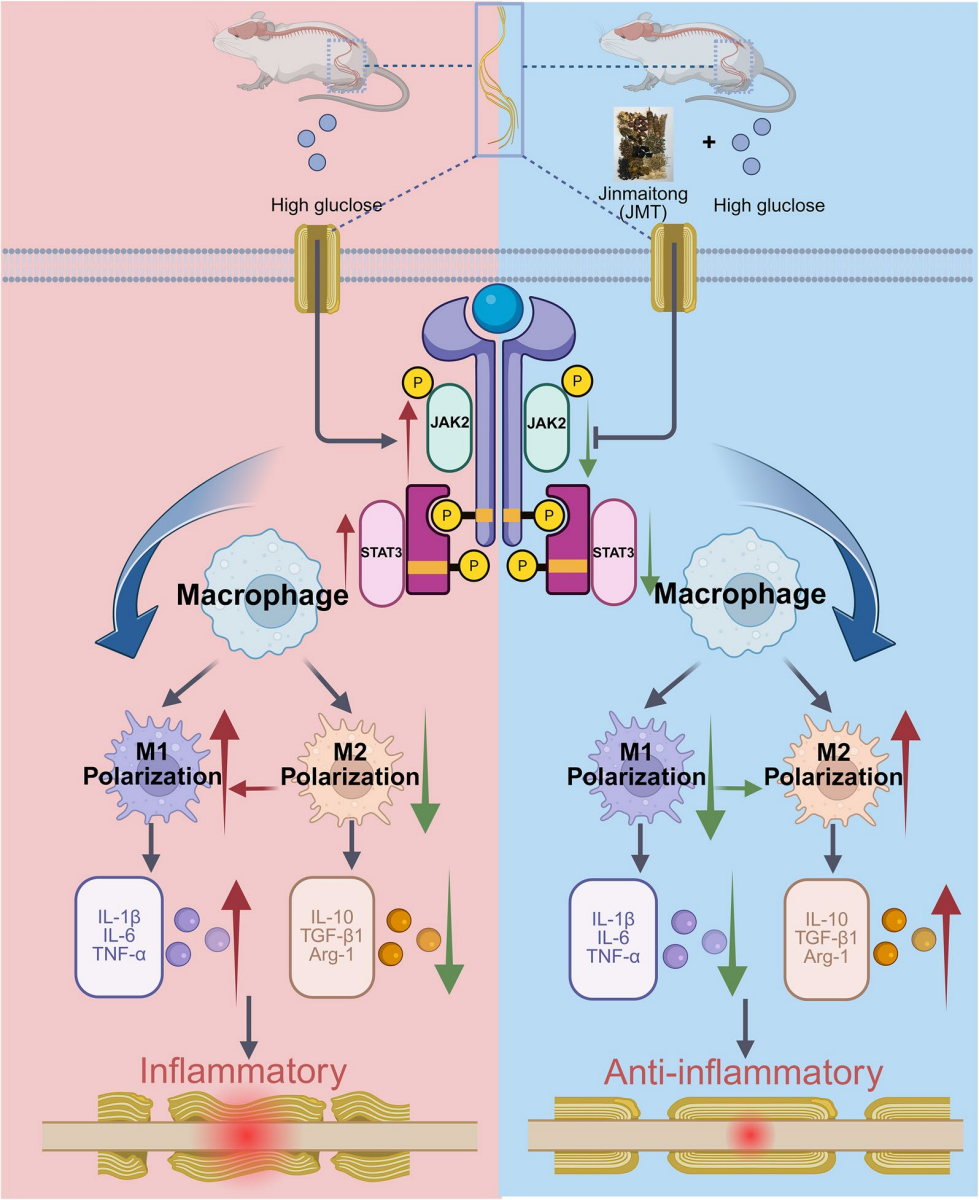

**Supplementary Information:**

The online version contains supplementary material available at 10.1186/s13020-025-01300-0.

## Introduction

Diabetes mellitus (DM) has become a global health burden with a steadily increasing prevalence [1]. Diabetic peripheral neuropathy (DPN) is one of the most common complications of DM and a major cause of morbidity and disability in affected patients [2]. Patients with DPN typically experience sensory loss, allodynia, and muscle weakness in the extremities, which can lead to amputation and, in severe cases, death [3]. Moreover, the prevalence of DPN has been reported to be higher in patients with type 2 diabetes mellitus (T2DM) than in those with type 1 diabetes mellitus (T1DM) [4].

The pathogenic mechanisms of DPN are complex and remain unclear. Systemic inflammation has been implicated in both the onset and progression of DPN [5]. Growing evidence has demonstrated that experimental animals with T2DM-induced neuropathy exhibited an increased inflammatory response, characterized by macrophage infiltration and proliferation in the sciatic nerve [6, 7]. Macrophages play a critical role in axonal regeneration following peripheral nerve injury [8]. Following peripheral nerve injury, the inflammatory response is primarily mediated by infiltrating macrophages, which are generally polarized into two opposing but complementary phenotypes: pro-inflammatory (M1) and anti-inflammatory (M2) [8]. M1 macrophages, typically induced by lipopolysaccharide (LPS), produce pro-inflammatory cytokines such as tumor necrosis factor alpha (TNF-α), interleukin-1 beta (IL-1β), and interleukin-6 (IL-6). Conversely, M2 macrophages, induced by interleukin-4 (IL-4), produce anti-inflammatory cytokines such as interleukin-10 (IL-10) [9]. Multiple studies have demonstrated that M1 macrophages are activated and express high levels of inflammatory cytokines under hyperglycemic conditions, thereby contributing to the development of DPN [10, 11]. The pro-inflammatory mediator IL-6 is closely associated with the pathogenesis of DPN. The inhibition of TNF-α has been shown to improve nerve conduction velocity, enhance nerve blood flow, and ameliorate abnormal axonal morphology in diabetic rats [12]. In addition, elevated levels of inflammatory cytokines, including TNF-α and IL-6, have been observed in both patients and rodent models of DPN [13]. Thus, inflammation is considered a potential contributor to the pathogenesis of DPN. Macrophage polarization is regulated by various molecular mechanisms. Previous studies have highlighted a strong association between macrophage polarization and the Janus kinase 2 / signal transducer and activator of transcription 3 (JAK2/STAT3) signaling pathway [14]. Reduction of IL-6 and interleukin-8 (IL-8) significantly inhibited M2 macrophage polarization via the JAK2/STAT3 signaling pathway [15].

Although strict glycemic control is the standard management strategy, effective treatments for DPN are still lacking due to its complex pathophysiology. Moreover, glycemic control alone is insufficient to prevent the onset and progression of DPN. According to Traditional Chinese Medicine (TCM) theory, the clinical manifestations of DPN fall under the category of TCM syndromes such as ‘Jinbi.’ The main pathogenesis involves prolonged diabetes consuming kidney yin, leading to yin deficiency with internal heat, which scorches body fluids, resulting in blood stasis that blocks the tendons and vessels [16]. Over time, yin deficiency affects yang, leading to cold congealing the blood and causing stagnation, which prevents qi and blood from reaching the four extremities, resulting in malnourishment of the muscles, tendons, and vessels.

JMT is an in-hospital preparation at Peking Union Medical College Hospital. It is a Chinese herbal compound developed based on this TCM theory, with the effects of ‘tonifying the kidney, activating blood circulation, and warming and dredging the tendons and vessels.’ Previous randomized controlled trials have shown that JMT can significantly alleviate symptoms such as cold extremities, numbness, and pain in DPN patients, improve abnormal neurological signs and indicators including pinprick sensation and nerve conduction velocity, with few adverse effects [17, 18]. Additionally, studies have demonstrated that JMT can reduce the expression of inflammatory factors IL-1β and IL-18 in the serum of diabetic rats, as well as inhibit the activation of thioredoxin-interacting protein and the NLRP3 inflammasome in the sciatic nerve [19]. However, the potential anti-inflammatory mechanisms of JMT in DPN remain unclear. This study aims to clarify whether JMT ameliorates DPN by modulating the JAK2/STAT3 signaling pathway, promoting macrophage polarization, and thereby suppressing neuroinflammation.

Therefore, we performed ultra-performance liquid chromatography coupled with high-resolution mass spectrometry (UPLC-HRMS) on JMT to identify the main components. The ‘Drug-Target-Pathway’ association network was established to predict the potential targets by network pharmacology analysis. Then we divided the experiments into two parts. In the first part, we investigated the effects of different doses and treatment durations of JMT on neurological function and inflammatory markers in DPN rats. By correlating these findings with the temporal dynamics of inflammation during DPN progression, we aimed to determine the optimal timing for further intervention with the JAK2/STAT3 pathway inhibitor AG490. Subsequently, we combined inhibitor experiments to further explore whether JMT alleviates DPN by modulating macrophage polarization via the JAK2/STAT3 signaling pathway.

## Materials and methods

### Drugs and reagents

According to our previous study [20], JMT consists of 12 kinds of herbal medicines, including Semen *Cuscutae,* Fructus *Ligustri Iucidi,* Herba *Ecliptae,* Herba *Prunella Vulgaris,* Semen *Litchi,* Ramulus *Cinnamoml,* Rhizoma *Corydalis,* Semen *Persicae,* Senmen *Cassiae, Radix et Rhizoma Asari, scorpio* and *hirudo* in a fixed ratio of 10: 10: 10: 10: 30: 10: 10: 10: 30: 3: 3: 3 (Fig. S1, Table S1). All crude drugs were provided by Beijing Tongrentang Pharmaceutical Co., Ltd. (Beijing, China) and verified by Prof. Liang Xiaochun (Peking Union Medical College Hospital, Beijing, China) according to the Chinese Pharmacopoeia. The mixed herbs were soaked in water for two hours, then decocted under reflux with 10 volumes of distilled water for two hours. After filtration, the residue was decocted again with eight volumes of distilled water under reflux. The combined filtrates were vacuum-concentrated and freeze-dried, with yield of 19%. The lyophilized JMT powder was stored at  − 20 °C and resuspended in distilled water before administration.

The other drugs, antibodies, and kits used are listed in Table S2.

### Component analysis of JMT

Exactly 100 mg of the aforementioned lyophilized JMT powder was weighed and homogenized with 300 μL of extraction solvent (methanol:water = 4:1, v/v) through sequential cryogenic grinding and ultrasonication under low-temperature conditions. The mixture was centrifuged at 13,000 × g for 15 min, and the supernatant was collected for analysis. Chromatographic separation was performed on a Thermo Scientific™ Q Exactive™ UHPLC-FTMS system equipped with an ACQUITY UPLC BEH C18 column (100 mm × 2.1 mm internal diameter, 1.7 μm; Waters, Milford, USA). The mobile phase gradient elution program is detailed in Table 1. An injection volume of 3 μL was used, with simultaneous detection in both positive and negative electrospray ionization (ESI) modes. System parameters were optimized to ensure high-resolution mass spectrometric performance throughout the entire analytical process. The chemical composition of JMT was profiled and shown in Table S4. A total of 70 compounds were putatively identified by comparing their high-resolution mass spectrometry data with literatures.

**Table 1:** Gradient elution

Time (min)	A (%)	B (%)
0	98	2
0.5	98	2
3.5	75	25
7.5	65	35
11	50	50
13	5	95
14.4	5	95
14.5	98	2
16	98	2

### Network pharmacology analysis

Preliminary identification of JMT’s chemical constituents was performed using UPLC-HRMS analysis combined with literature reports. The identified JMT components were submitted to the SwissADME database (http://www.swissadme.ch/index.php) to obtain information on their physicochemical properties, pharmacokinetics, drug-likeness, and medicinal chemistry friendliness. Compounds exhibiting high gastrointestinal absorption (GA) and satisfying drug-likeness (DL) criteria for at least two out of five filters (Lipinski, Ghose, Veber, Egan, Muegge) were selected as the active constituents of JMT.

These active JMT constituents were imported into the SwissTargetPrediction database (http://www.swisstargetprediction.ch/), with Homo sapiens selected as the species. Targets with a probability > 0.0 were included as potential targets related to JMT’s active ingredients. Concurrently, disease targets associated with DPN were collected from the GeneCards and OMIM databases. Common targets between JMT and DPN were identified through Venn diagram analysis.

To explore molecular mechanisms, the common targets were imported into the STRING platform (https://string-db.org) to construct a Protein–Protein Interaction (PPI) network. The resulting interaction network was analyzed and visualized using Cytoscape 3.8.0. We screened for core genes by applying automatically generated thresholds to three network parameters. The thresholds were as follows: degree centrality (DC) ≥ 39.49, closeness centrality (CC) ≥ 0.00097, and betweenness centrality (BC) ≥ 575.30. Node degree values were calculated, with color intensity and node size proportional to the degree value or target significance.

Functional enrichment analysis was conducted using the DAVID database (https://david.ncifcrf.gov/). Gene Ontology (GO) terms were sorted by ascending p-value, with the top 10 significantly enriched terms selected for analysis. Kyoto Encyclopedia of Genes and Genomes (KEGG) pathways were similarly sorted by ascending p-value, retaining the top 20 pathways (excluding entries related to human diseases). A gene-pathway interaction network was constructed to elucidate the relevant regulatory mechanisms.

Molecular docking was performed on the top six active ingredients screened by JMT with the proteins JAK2 and STAT3. The protein structures were retrieved from the PDB database (https://www.rcsb.org), and the 2D molecular structures were obtained from PubChem (https://pubchem.ncbi.nlm.nih.gov). CB-Dock (https://cadd.labshare.cn/cb-dock2/index.php) was used to automatically detect potential binding cavities and perform blind docking [21]. The docking was carried out using AutoDock Vina. Key parameters, including the size and center of the search box, are automatically determined by CB-Dock using its built-in, validated curvature-based cavity detection algorithm. A binding energy of ≤ -5.0 kcal/mol is used as the threshold to determine significant binding activity between the ligand and the target. All docking experiments were independently run multiple times, and the results were visualized [22].

### Animal experiment design

The study was approved by the Institutional Animal Care and Use Committee of Peking Union Medical College Hospital (Approval No. XHDW-2022–015, February 2022), and the experimental procedures were in accordance with the Guidelines for the Keeping and Use of Laboratory Animals issued by the Animal Research Council of China.

Male Sprague–Dawley (SD) rats aged 4–6 weeks (weighing 200–250 g) were purchased from SPF (Beijing) Biotechnology Co., Ltd. (Beijing, China) and housed in a specific pathogen-free (SPF) environment at the Experimental Animal Center of Peking Union Medical College Hospital. After one week of adaptive feeding, all rats were fed with high fat diet (HFD; 67% basal feed, 20% sucrose,10% lard,2.5% cholesterol and 0.5% sodium cholate, Beijing Keao Xieli Feed Co.,Ltd.). After six weeks of HFD, a single dose of 35 mg/kg streptozotocin (STZ) dissolved in 10 mmol/L citrate buffer (pH 4.5) was injected intraperitoneally into the HFD-fed rats [23]. Random blood glucose was measured with the blood from tail tip after 72 h. Rats with blood glucose ≥ 16.7 mmol/l were considered successful induction of T2DM. Rats were randomly assigned to groups (n = 6 per group) using a random number table. The experimenters in charge of conducting the experiment and data analysis were blinded and unaware of group allocations.

**Fig. 1:**
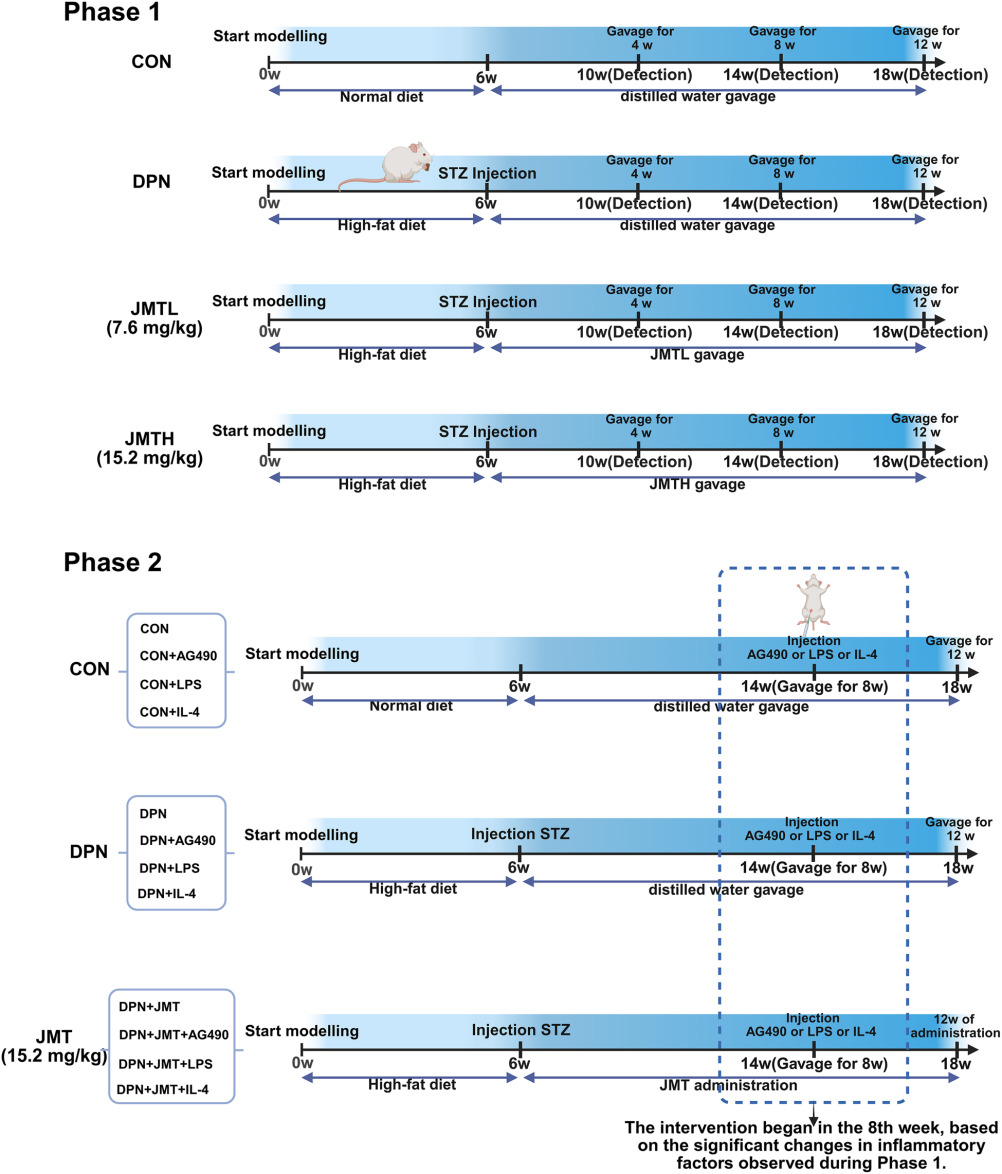
Schematic diagram of rat grouping, modelling and treatment

In the first batch, the rats were divided into the healthy control group (CON), the T2DM rats with DPN group (DPN), the DPN rats treated with low-dose JMT (7.6 mg/kg) (equal to clinical dose, based on the weight of JMT lyophilized powder) group (DPN + JMTL), and the DPN rats treated with high-dose JMT (15.2 mg/kg) group (DPN + JMTH) [24]. The CON group and DPN group received the same volume of distilled water, and the other groups received JMT gavage administered daily (Fig. 1). To observe the effect of JMT on DPN and the change of inflammation in DPN at different time points, the rats in each group were sacrificed four, eight, twelve weeks after diabetes induction respectively.

Subsequently, the second batch of experiments was conducted to investigate the potential mechanism of JMT against DPN. To further explore the relationship between JMT and the JAK2/STAT3 pathway as well as macrophage polarization, rats were administered the JAK2/STAT3 signaling pathway inhibitor AG490 (20 mg/kg) [25], the M1 macrophage polarization agonist LPS (0.5 mg/kg) [26] and the M2 macrophage polarization agonist IL-4 (1.5 mg/kg) [27]. AG490, LPS, or IL-4 was dissolved in dimethyl sulfoxide and saline, and injected three times per week. The rats were randomly divided into twelve groups, as shown in Fig. 1: CON, DPN, DPN + JMT, CON + AG490, DPN + AG490, DPN + JMT + AG490, CON + LPS, DPN + LPS, DPN + JMT + LPS, CON + IL-4, DPN + IL-4, DPN + JMT + IL-4.

After administration for 12 weeks, all rats were anesthetized with 2% isoflurane (0.41 mL/min fresh gas flow rate of four L/min), and whole blood samples were collected. The sciatic nerve and bone marrow were collected for further experiments.

### Neurological function assessments

#### Mechanical threshold

Mechanical thresholds were detected using the von Frey pain measurement instrument (IITC Life Science Inc., Woodland Hills, CA, USA) as previously described [28]. The probe tip of the instrument was gently applied upwards to the plantar surface of the rat’s mid-foot, and the force was then applied slowly until the rat withdraw, flicked, or licked its paw. The reading of the maximum force (in grams) was automatically recorded.

#### Thermal response latency

To determine the sensitivity of rats to noxious heat, the hot-plate test and tail flick test were conducted. In the hot-plate test, rats were placed individually on a hot plate apparatus (UGO Basile Inc., Varese, Italy) of which temperature was set at 50 ± 0.2 °C [29]. A positive response was defined as withdrawal or retraction of the hind paw. Then, the latency was recorded. In the tail flick test, the middle of the rat tail was placed on the tail flick apparatus (UGO Basile Inc., Varese, Italy), the intensity of the heat stimulus was adjusted to 50% and the latency of the tail retraction response was measured [30]. Individual measurements were repeated three times, and the mean value was calculated as the thermal response latency.

#### Motor nerve conduction velocity (MNCV) measurement

MNCV was measured using a bioelectric amplifier (PLC01PowerLab C, FE238 Octal Bio Amp, Australia). After anesthetizing the rat, the sciatic nerve was exposed following the method described in the literature [31]. Subsequently, the stimulating electrode S1 was inserted into the proximal site of sciatic nerve. The recording S2 was inserted into the distal site. The distance D between S1 and S2 was recorded. The ground electrode was placed at the root of rat tail. After an electrical stimulus, the latency T was obtained. The MNCV was calculated by dividing the distance D by the latency T.

### Sample collection

Blood was collected from the abdominal aorta after anesthesia. Blood samples were centrifuged at 3000 r/min for 10 min, then serum was separated and stored at −80 °C for enzyme-linked immunosorbent assay (ELISA). The sciatic nerve on both sides of the rat was separated. The left sciatic nerve was cut into two segments. One segment (3–4 mm) was fixed in 2.5% glutaraldehyde at 4 °C for transmission electron microscopy (TEM). The other segment was fixed in 4% paraformaldehyde (PFA) for pathological examination. The right sciatic nerve was divided into two segments after measuring MNCV and stored at −80 °C for quantitative polymerase chain reaction (qPCR) and Western blot. The femur was separated and stored in PBS for bone marrow extraction preparing for flow cytometry.

### Hematoxylin and Eosin (H&E) staining

The sciatic nerves were immersed in 4% PFA for 24 h for fixation. Subsequently, the samples were dehydrated with graded concentrations of ethanol and embedded in paraffin, then cut into sections of four-μm-thick sections. The slides were deparaffinized in xylene, followed by rehydration through a graded series of ethanol.

Next, the sections were stained with hematoxylin for five minutes. Differentiation was performed in 1% acid alcohol for 10 s, followed by bluing in 0.2% ammonia water for one minute. Subsequently, the sections were counterstained with eosin for one minute, then dehydrated through ascending concentrations of ethanol. The slides were cleared in xylene for 10 min and sealed with neutral gum. The images were finally acquired and analyzed using a slide scanner (Pannoramic250, 3D HISTECH, Hungary).

### Luxol fast blue (LFB) myelin staining

LFB myelin staining was performed with LFB staining kit according to the manufacturer’s protocol. The sections of sciatic nerve were stained with Fast Blue myelin staining. The slices were observed under a slide scanner, and the images were captured and analyzed.

### TEM examination

Tissue samples were harvested and immediately fixed in 2.5% glutaraldehyde at 4 °C for 24 h. Then, the post-fixation was performed in 1% osmium tetroxide for one hour at room temperature. Next, the tissues were dehydrated through a graded ethanol series. The samples were then treated with propylene oxide as a transitional solvent before embedding in epoxy resin. Following infiltration with a 1:1 mixture of propylene oxide and epoxy resin for one hour, the tissues were embedded in 100% epoxy resin and polymerized in an oven at 60 °C for 48 h. Ultrathin Sects. (60–90 nm) were cut and mounted on copper grids. The sections were contrasted with 2% uranyl acetate for 10 min and lead citrate for five minutes. The sections were examined using a transmission electron microscope (TEM-1400, JEOL, Tokyo, Japan). Morphometric parameters such as the proportion of abnormal fibers (irregularly shaped fibrous or compressed myelin sheaths) and the G-ratio (the ratio of the inner diameter of the axon to the outer diameter of the myelin sheath) were analyzed using ImageJ software (version 1.46r; National Institute of Health, Bethesda, MD, USA).

### ELISA

The levels of IL-1β, monocyte chemoattractant protein-1 (MCP-1), inducible nitric oxide synthase (iNOS), IL-10, and transforming growth factor-beta 1 (TGF-β1) in serum of rats were measured using ELISA kits according to the manufacturer’s instructions. In brief, standards or samples are added to the microplate pre-coated with the antibodies. After incubation and washing, biotinylated detection antibodies are added. Following a second incubation and washing, HRP enzyme conjugate is added. After incubation, substrate solution is introduced, and the reaction is stopped by adding stop solution. Finally, the optical density (OD) is measured at the wavelength of 450 nm using a microplate reader (Synergy H1, Thermo Fisher, USA). A standard curve is generated by plotting the OD values of the standards against their known concentrations, and the sample concentrations are calculated based on this curve.

### qPCR

Total RNA was extracted from the sciatic nerve using Trizol reagent, and cDNA was synthesized from the RNA using a PrimeScript RT kit according to the manufacturer’s instructions. cDNA was detected using SYBR Green PCR Master Mix for PCR on a real-time PCR detection system (Thermo Fisher QuantStudio 7Pro, Massachusetts, USA). ACTB (β-actin) was used as an internal control. Primers used in this study are shown in Table S3.

### Flow cytometry

Following euthanasia of the rats, femurs and tibias were dissected. Bone marrow tissue was flushed with DMEM medium. After lysis of red blood cells, the cells were cultured with DMEM containing with 15% fetal bovine serum (FBS) and 10 ng/mL macrophage colony-stimulating factor (M-CSF) for seven days. Subsequently, the cells were identified as bone marrow-derived macrophages (BMDMs) by flow cytometry analysis. The cells resuspended with ice-cold PBS. Following centrifugation (2000 rpm × 10 min), the supernatant is discarded. A volume of 100 μL of flow cytometry fixation buffer (PBS containing 0.5% BSA) is added to each microcentrifuge tube. The samples are then vortexed vigorously and washed once or twice. Finally, the cells are resuspended in 100 μL of fixation buffer and stained with Zombie Aqua™ dye. Subsequently, 10% goat serum was added for blocking, and the cells were incubated in the dark at 4 °C for 30 min with the following antibodies: CD45-APC-eFluor 780, CD11b-APC, CD86-FITC, and CD163-PE. Finally, the stained cells were analyzed using a flow cytometer (Attune Nxt 3L-BRV, Thermo Fisher, USA), and the data were processed with FlowJo 10 software (Becton, Dickenson and Company, OR, USA). As shown in Fig. S3, after excluding cell doublets (based on FSC-A/FSC-H) to gate the single cell population (singlets) and selecting viable cells, CD45⁺CD11b⁺ double-positive cells were gated as macrophages. Subsequently, based on the expression of their surface markers, CD86⁺ cells were defined as M1 macrophages, while CD163⁺ cells were defined as M2 macrophages.

### Immunofluorescence

The paraffin-embedded sciatic nerve slices were deparaffinized and dehydrated through a graded ethanol series. After antigen retrieval, the slices were blocked with 10% goat serum. The slices were incubated with the following primary antibodies at 4 °C overnight: NF160, S100, growth associated protein 43 (GAP43), CD86, CD163, followed by incubation with species-matched fluorescent secondary antibodies at room temperature for one h. All images were captured by a laser scanning confocal microscope (Nikon Inc., Tokyo, Japan) and analyzed using ImageJ software.

### Western blot

Total protein of sciatic nerves was extracted by centrifugation after being lysed by RIPA lysis buffer which contained protease inhibitor and phosphatase inhibitor. The protein concentration was determined using a BCA protein kit following the manufacture’s instruction. The extraction was mixed with 5 × loading buffer and heated at 95 °C for 10 min. Then, the proteins were separated by SDS–polyacrylamide gel electrophoresis and transferred to a PVDF membrane. After blocking, the membrane was incubated with the following primary antibody overnight at 4 °C: rabbit anti-IL-1β (1:1000), rabbit anti-IL-6 (1:1000), rabbit anti-TNF-α (1:1000), rabbit anti-Arginase-1 (Arg-1) (1:1000), rabbit anti-TGF-β1 (1:1000), rabbit anti-IL-10 (1:1000), rabbit anti-p-JAK2 (1:1000), rabbit anti-JAK2 (1:1000), rabbit anti-p-STAT3 (1:1000), and rabbit anti-STAT3 (1:1000). After washing with TBST, the blot was incubated with the corresponding secondary antibody for one hour at room temperature. The protein band was visualized by the ultrasensitive chemiluminescence reagent. The gray scale value of the protein band was analyzed using ImageJ software (version 1.46r; National Institute of Health, Bethesda, MD, USA).

### Statistical analysis

All data are expressed as mean ± standard deviation (SD). Multiple comparisons were performed using ordinary one-way analysis of variance (ANOVA). *P* < 0.05 was considered significant. Images and blots were quantified using ImageJ software. The statistical analyses were performed using Statistical Package for the Social Sciences (Spss) program (version 23.0; IBM Corp., Armonk, NY, USA) and visualization were performed using GraphPad Prism software (version 9.3.1; Bethesda, MD, USA).

## Results

### High-dose JMT treatment improved peripheral nerve function and suppressed inflammation of DPN rats

**Fig. 2:**
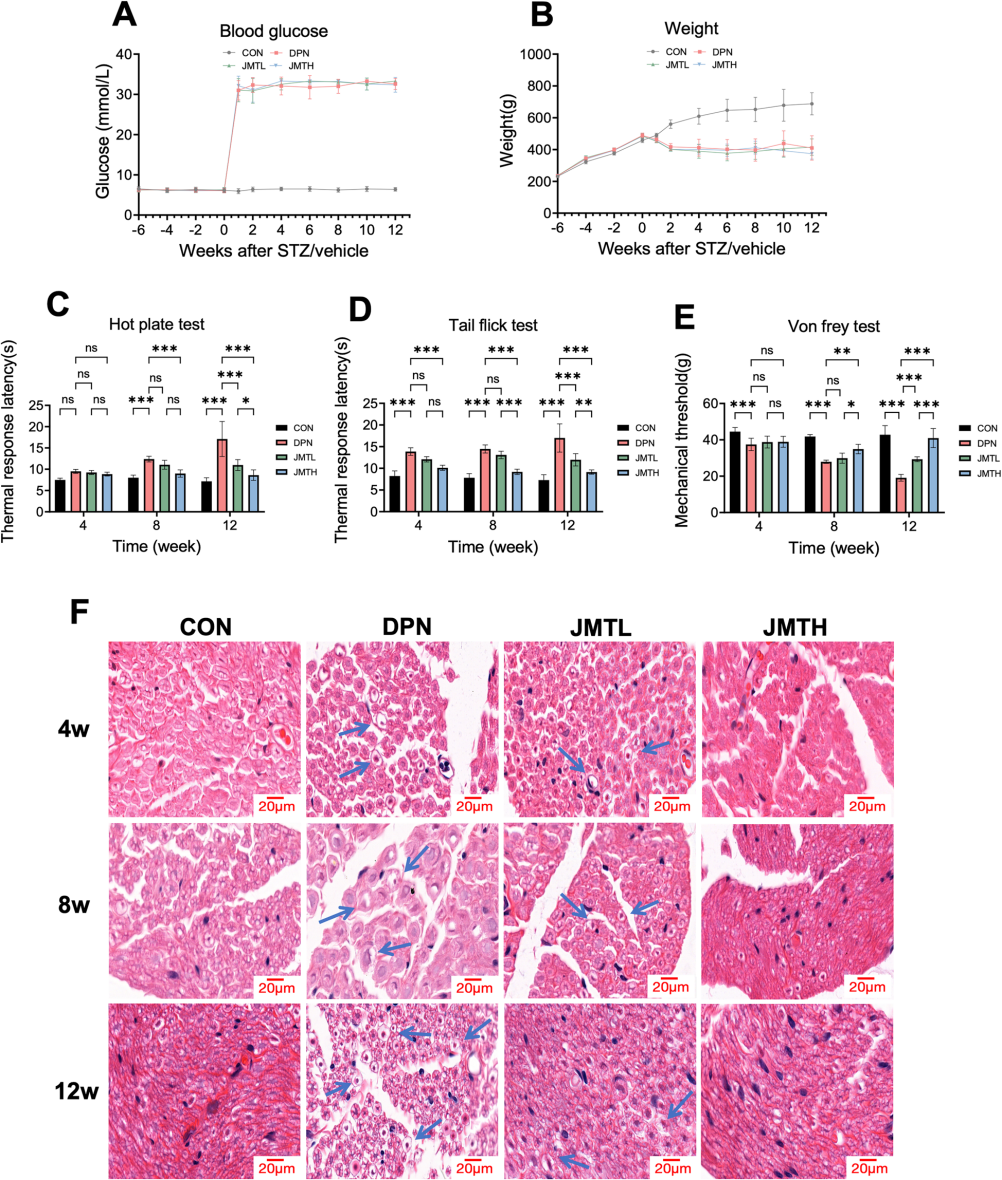
The neuroprotective effect of JMT on DPN rats. **A**, **B** Blood glucose levels (**A)** and body weight (**B)** were monitored in different groups during the experiment. **C**, **D** The thermal response latency detected by the tail flick test (**C)** and the hot plate test (**D**). **E** The mechanical threshold detected by the von Frey test. **F** H&E staining of the cross-section of the rat sciatic nerve (scale bar = 20 µm). Data were shown as mean ± SD (n = 6). *P < 0.05, **P < 0.01, ***P < 0.001, *ns*  not significant

In the first batch of experiment (Batch 1), following six weeks of HFD feeding and a single intraperitoneal injection of STZ, the blood glucose levels of rats exceeded 16.7 mmol/L, thereby indicating the successful establishment of the T2DM model (Fig. 2A). The blood glucose levels of diabetic rats continuously increased, while their body weight significantly decreased when compared to the healthy controls one week after diabetes induction (Fig. 2A, B). Two different doses of JMT were administrated to the diabetic rats for four, eight and twelve weeks. However, no significant differences in blood glucose levels or body weight were observed among the DPN group, the JMTL group and the JMTH group at different time points.

As shown in Fig. 2C, E, four weeks after diabetes induction, reduced mechanical pain thresholds were observed in rats via the von Frey test, and prolonged thermal response latency was measured by the tail-flick test, while no differences were observed among the groups in the hot plate test, and JMT did not demonstrate significant therapeutic effects. Starting from the eighth week, increased thermal latency was observed in the hot plate test, suggesting that the tail-flick test may be more sensitive for thermal nociceptive assessment. High-dose JMT treatment for eight weeks significantly improved both mechanical pain thresholds and thermal response latency in DPN rats. After 12 weeks of treatment, both the JMTL and JMTH groups exhibited substantial improvements in peripheral nerve function, with the effects displaying dose- and time-dependent characteristics.

Histological analyses of the sciatic nerve revealed progressive neurodegenerative changes in untreated DPN rats, including myelinated fiber swelling, axonal atrophy, and vacuolar degeneration (Fig. 2F). These pathological alterations were markedly alleviated by JMT treatment, with JMTH exhibiting significantly greater therapeutic efficacy compared to JMTL.

**Fig. 3:**
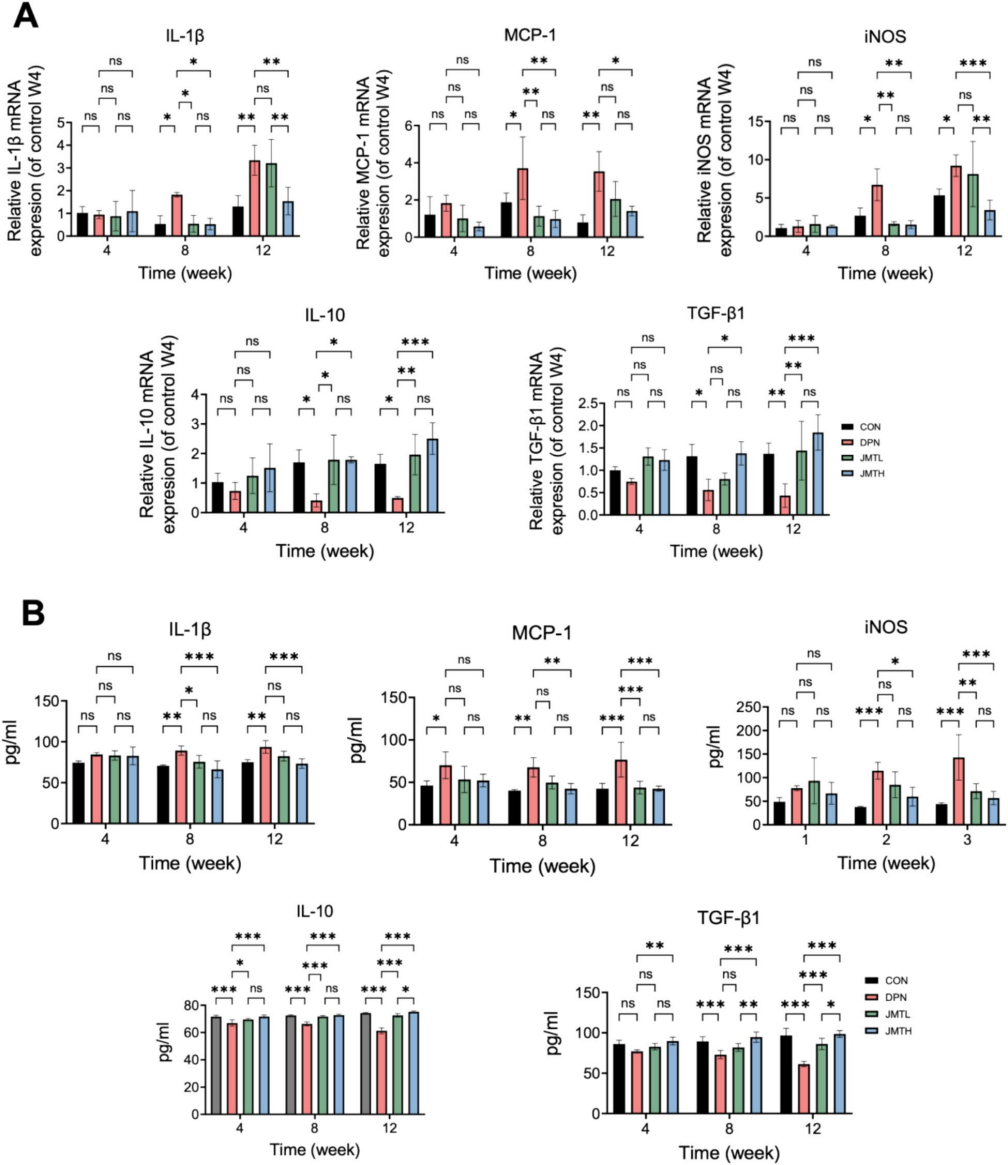
The effects of JMT on inflammatory cytokines in DPN rats. **A** Pro-inflammatory factors IL-1β, MCP-1, iNOS, and anti-inflammatory factors IL-10, TGF-β1 mRNA in the sciatic nerve detected by qPCR. **B** Pro-inflammatory factors IL-1β, MCP-1, iNOS, and anti-inflammatory factors IL-10, TGF-β1 in serum detected by ELISA. Data were shown as mean ± SD (n = 3). *P < 0.05, **P < 0.01, ***P < 0.001, *ns*  not significant

Four weeks after diabetes induction, no significant inter-group differences were observed. This absence of disparity might be attributable to the early diabetic stage, where inflammatory manifestations were not yet present, and the short duration of the JMT intervention. At the 8-week time point following diabetes induction, the DPN group exhibited significantly elevated levels of pro-inflammatory cytokines (iNOS, MCP-1, IL-1β) and reduced levels of anti-inflammatory cytokines (IL-10, TGF-β1) in both serum (Fig. 3A) and sciatic nerve tissues (Fig. 3B), compared to the control group. Based on these inflammatory changes, the 8-week mark was selected as the optimal intervention point for administering AG490, LPS, and IL-4 in the second phase of the experiment to explore the molecular mechanisms underlying DPN. Notably, treatment with JMT reversed these alterations in a dose-dependent manner, with the high-dose group demonstrating greater efficacy than the low-dose group.

### Chemical composition of JMT

**Fig. 4:**
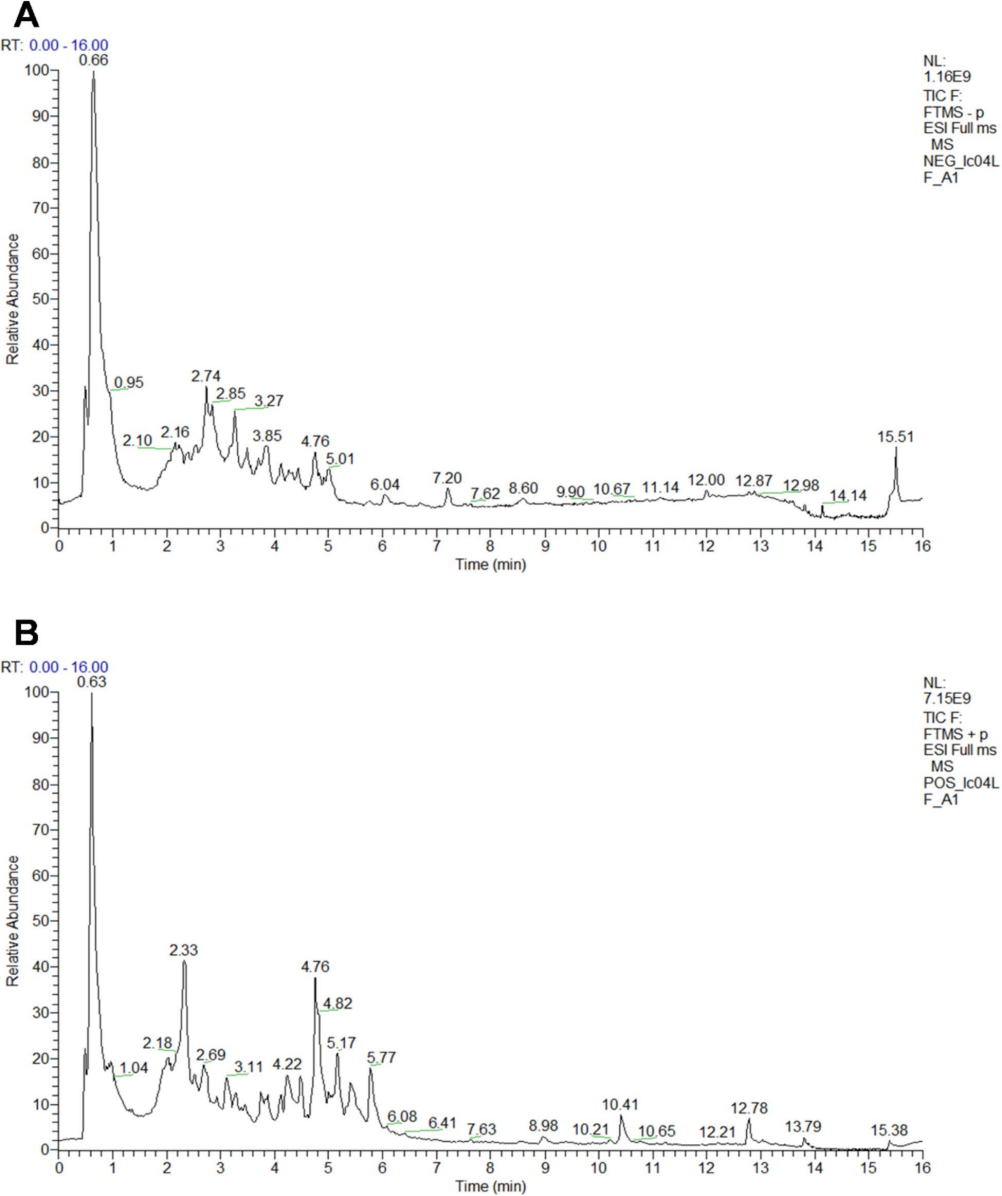
Chemical composition of JMT profiled by ultra-performance liquid chromatography-high resolution mass spectrometry (UPLC-HRMS) analysis. **A** Positive ion mode. **B** Negative ion mode

This study employed UPLC-HRMS analysis to characterize the chemical profile of JMT. The total ion chromatograms (TICs) of JMT in both positive and negative ion modes are presented in Fig. 4A, B. By comparing the high-resolution mass spectrometry data with literature reports [32–41]. The identified compounds encompass most of the principal chromatographic peaks and exhibit structural diversity. 70 compounds were tentatively identified, including 4-Coumaric acid, Aurantio-obtusin beta-D-glucoside, Formononetin, Quercetin, Loliolide, L-phenylalanine and so on (Table S4). Notably, major chemical classes in JMT included flavonoids, phenolic acids, and alkaloids and their derivatives (Fig.S4).

### Network pharmacology analysis

**Fig. 5:**
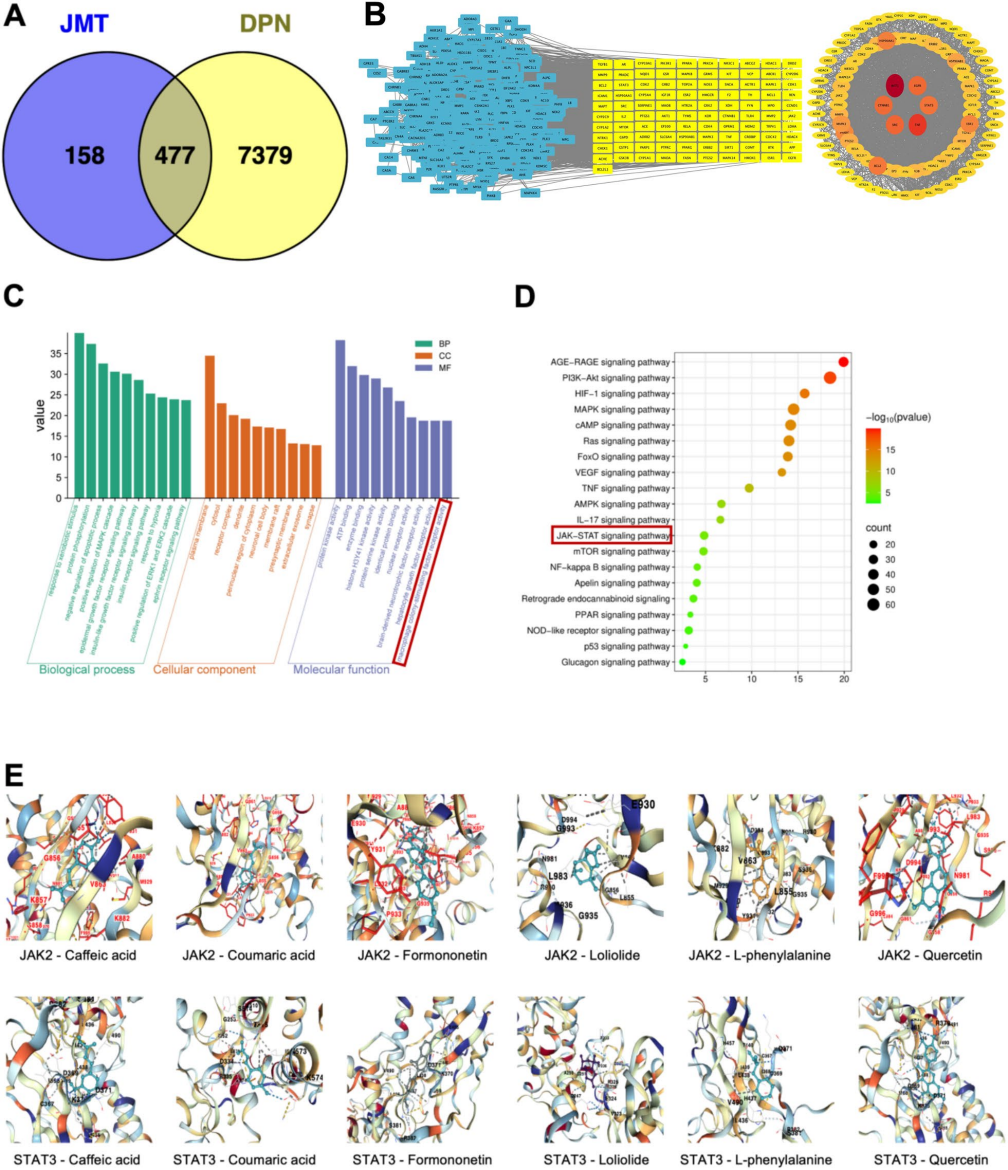
Network-based pharmacology exploring the mechanisms by which JMT improves DPN. **A** Venn diagram of cross-targets between JMT and DPN. **B** PPI network of cross-targets between JMT and DPN. **C** GO enrichment analysis. **D** KEGG pathway analysis. **E** Molecular docking of the main compounds of JMT with JAK2 and STAT3

Based on the aforementioned compounds identified from JMT (Supplementary Table S4), we further determined 635 potential therapeutic targets of JMT by integrating protein- and peptide-associated gene data from SwissTargetPrediction platform following screening via SwissADME database (Table S4). Disease-associated targets were retrieved from two public databases, yielding 7856 candidate genes. Venn diagram analysis revealed 477 overlapping targets between JMT components-related and DPN-related genes (Fig. 5A). These overlapping targets were analyzed using the STRING database to construct a PPI network. Subsequently, Cytoscape 3.7.2 was employed to integrate JMT components with shared genes, ultimately visualizing the comprehensive interaction network (Fig. 5B). The left panel displays a filtered cluster of core genes, while the right panel presents the consolidated hub genes—notably including AKT1, TNF, EGFR, STAT3, and JAK2 as central regulators.

GO functional enrichment analysis of the shared genes revealed that the therapeutic mechanism of JMT against DPN primarily involves the molecular function of macrophage colony-stimulating factor receptor activity (Fig. 5C). KEGG pathway analysis identified critical signaling pathways associated with JMT’s therapeutic effects against DPN, prominently featuring the TNF signaling pathway, IL-17 signaling pathway, and the JAK/STAT pathway—a central focus of this investigation (Fig. 5D). These pathways are implicated in inflammatory regulation. The observed shifts in pro-inflammatory and anti-inflammatory factor levels in prior experiments further demonstrate JMT’s ability to modulate macrophage polarization in DPN rat models, highlighting its immunoregulatory role in mitigating neuroinflammation. The JAK2/STAT3 signaling pathway, a key regulator of macrophage polarization [14], likely mediates this process.

This study assessed the interactions between the top six bioactive compounds of JMT (selected via SwissADME database) and the core targets JAK2 and STAT3 using molecular docking techniques (Fig. 5E). The results demonstrated that all 12 compound-target pairs exhibited binding energies below −5 kcal/mol (Table 2), suggesting strong binding capabilities between the primary compounds and target proteins [42]. Among these, formononetin and quercetin demonstrated significantly stronger binding affinities than other bioactive compounds, as indicated by their lower binding energy values.

**Table 2:** Binding energy of compounds to target proteins (kcal/mol)

	4-Coumaric acid	Formononetin	Quercetin	Loliolide	L-phenylalanine	Caffeic acid
JAK2	− 5.4	−8.4	−8.5	−6.1	−5.8	−5.3
STAT3	−5.6	−7.2	−7.6	−5.7	−5.2	−6.0

### JMT Ameliorated peripheral nerve function in DPN Rats partially through the JAK2/STAT3 signaling pathway

According to the literature [43], JAK2/STAT3 can influence inflammation by promoting macrophage polarization. Based on our network pharmacology analysis (Fig. 5), the potential therapeutic mechanism by which JMT alleviates inflammation in DPN may involve macrophage polarization and the dynamic regulation of the JAK/STAT signaling pathway. To further investigate the effects of JMT on the JAK2/STAT3 pathway and macrophage polarization, the second batch of experiments was conducted in combination with the corresponding inhibitor. Based on the results of batch 1 (Fig. 3), JMT significantly reduced the mRNA levels of inflammatory factors in DPN rats starting from the eighth week of treatment. Therefore, to further explore the molecular mechanisms by which JMT influences macrophage polarization, the eighth week was selected as the intervention time point for the second batch of the experiment, during which the JAK2/STAT3 inhibitor AG490 was administered via intraperitoneal injection. Additionally, to explore the mechanism by which JMT inhibits M1 macrophage polarization and promotes M2 macrophage polarization, we administered the M1 macrophage polarization agonist LPS and the M2 macrophage polarization agonist IL-4 via intraperitoneal injection following eight weeks of JMT treatment. Furthermore, the optimal JMT intervention dose and duration were determined to be 15.2 mg/kg for 12 weeks, based on batch 1 (Figs. 2 and 3) and previous studies [24].

**Fig. 6:**
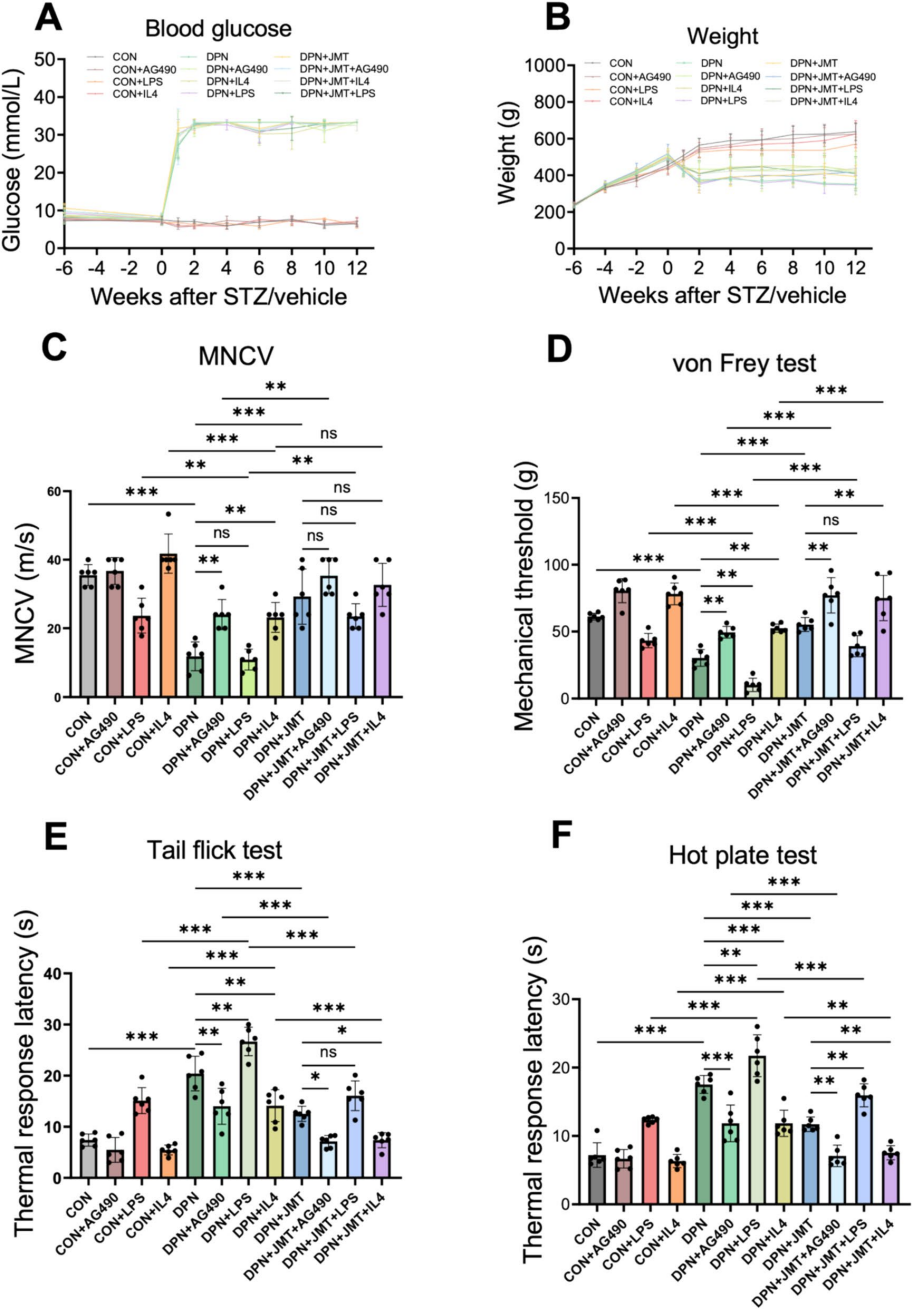
High-dose JMT treatment for 12 weeks significantly improved the neurological function in DPN rats. **A** Blood glucose. **B** Body weight. **C** MNCV. **D** Mechanical threshold detected by the von Frey test. **E**, **F** Thermal response latency detected by the tail flick test (**E**) and the hot plate test (**F**). Data were shown as mean ± SD (n = 6). *P < 0.05, **P < 0.01, ***P < 0.001, *ns*  not significant

In the second batch, the trends in blood glucose and body weight changes observed in the normal group, the DPN group and the JMT-treatment group were consistent with those observed in batch 1. Meanwhile, the administration of AG490, LPS, or IL-4 did not significantly affect the blood glucose or body weight in either DPN rats or healthy rats (Fig. 6A, B). At the 12th week after modeling, diabetic rats exhibited reduced MNCV, decreased mechanical threshold and prolonged thermal response latency (Fig. 6C–F).

After 12 weeks of treatment, JMT, AG490 and IL-4 significantly improved the behavioral test outcomes in DPN rats. However, LPS notably weakened the effect of JMT on thermal response latency, as measured by the hot plate and tail flick test. Additionally, while MNCV and mechanical threshold (von Frey test) showed a slight decrease in JMT-treated DPN rats following co-treatment with LPS, the differences were not statistically significant.

### JMT ameliorates sciatic nerve pathology in DPN rats via modulation of the JAK2/STAT3 pathway

#### Histological assessment via HE and LFB staining

**Fig. 7:**
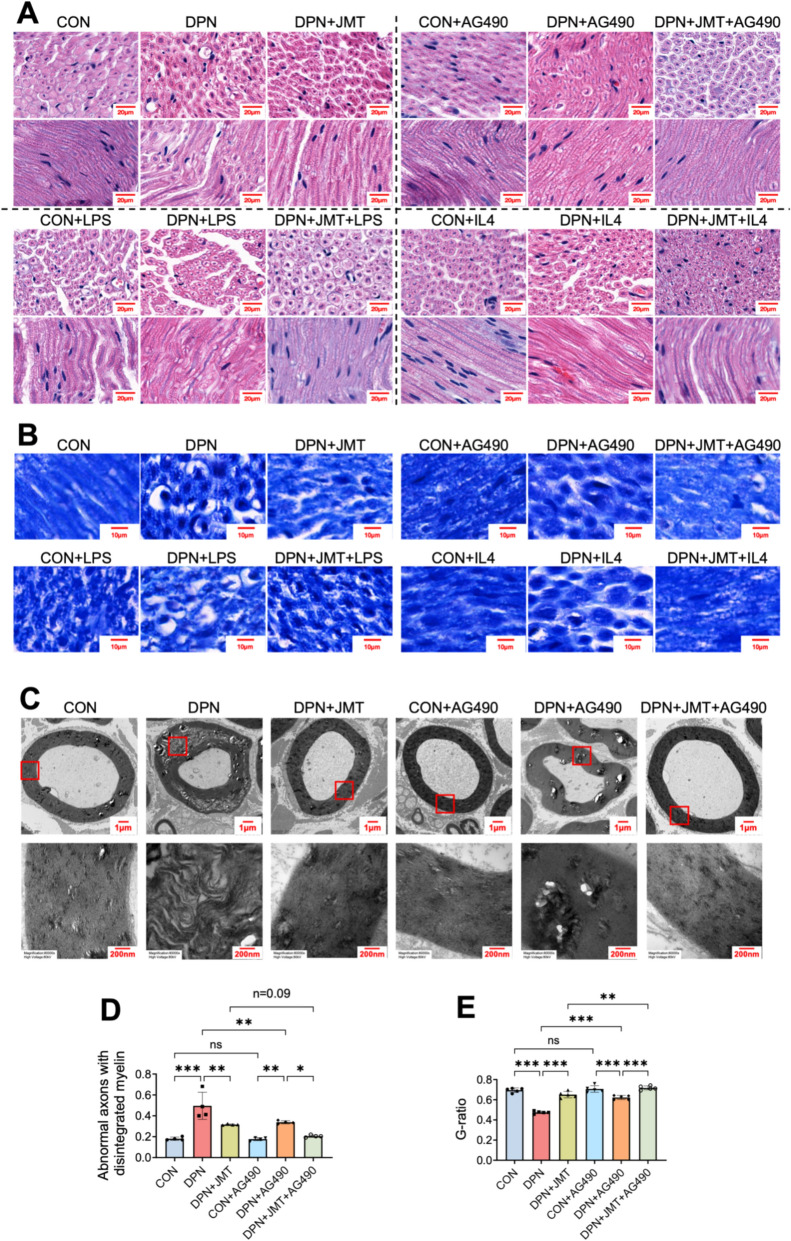
JMT treatment ameliorates pathomorphological alterations in the sciatic nerve of DPN rats. **A** H&E staining (scale bar = 20 µm). **B** LFB staining (scale bar = 10 µm). **C** The ultrastructure of the sciatic nerve observed by TEM (magnification:12,000 × , scale bar = 1 µm); the lower panel shows an enlarged image from the red box in the upper panel (magnification: 80,000 × , scale bar = 200 nm). **D**, **E** The percentage of abnormal axons with disintegrated myelin, and the G-ratio (axon diameter / myelin diameter)

To evaluate the neuroprotective effects of JMT in DPN rats through potential modulation of macrophage polarization via the JAK2/STAT3 signaling pathway, we performed histological assessments using H&E and LFB staining. In the DPN group, H&E staining (Fig. 7A) revealed disorganized sciatic nerve fibers, fusion and swelling of myelin sheaths and axons, as well as the presence of vacuolar degeneration—features not observed in the CON group. LFB staining (Fig. 7B) of the CON group demonstrated intense blue coloration, indicating dense, intact myelin sheaths and well-organized myelinated fibers. Conversely, the DPN group showed pale or diminished blue staining, suggestive of demyelination or myelin degradation. Treatment with JMT, AG490 (a JAK2/STAT3 inhibitor), or IL-4 (an M2 macrophage polarization inducer) alleviated pathological features, evidenced by better-aligned nerve fibers, more uniform axonal and myelin diameters, and milder lesion severity. Notably, co-treatment with JMT and either AG490 or IL-4 produced greater improvements, whereas LPS (an M1 polarization inducer) exacerbated nerve injury and attenuated the neuroprotective effects of JMT.

### Ultrastructural observation via TEM

TEM revealed well-organized, tightly packed lamellar myelin structures and intact axons in the sciatic nerve of control rats (Fig. 7C). In contrast, DPN rats exhibited marked ultrastructural abnormalities, including myelinolysis, large areas of lamellar separation, disorganization, and evident axonal edema. These pathological changes were substantially alleviated following treatment with JMT or AG490, with the most pronounced recovery observed in the JMT + AG490 co-treatment group.

### Quantitative morphological analysis

Morphometric analysis further confirmed the structural alterations (Fig. 7D, E). The proportion of abnormal axons was significantly elevated in DPN rats relative to controls. Additionally, the G-ratio—defined as the ratio of the axonal diameter to the total diameter including myelin—was markedly reduced in the DPN group, indicating axonal atrophy and myelin swelling. Treatment with JMT or AG490 significantly restored the G-ratio and decreased the number of abnormal axons.

### Modulation by JAK2/STAT3 inhibition and macrophage polarization

To further elucidate the involvement of JAK2/STAT3 signaling and macrophage polarization in JMT-mediated nerve protection, we utilized AG490 (pathway inhibition), IL-4 (M2 polarization), and LPS (M1 polarization) as modulators. The results demonstrated that both JMT and AG490 independently conferred structural protection to the sciatic nerve. IL-4 exhibited a similar protective profile, reinforcing the role of M2 macrophages. In contrast, LPS aggravated pathological changes and diminished the protective efficacy of JMT. These findings support that JMT’s beneficial effects may be mediated, at least in part, by promoting M2 macrophage polarization and attenuating JAK2/STAT3-driven inflammation.

**Fig. 8:**
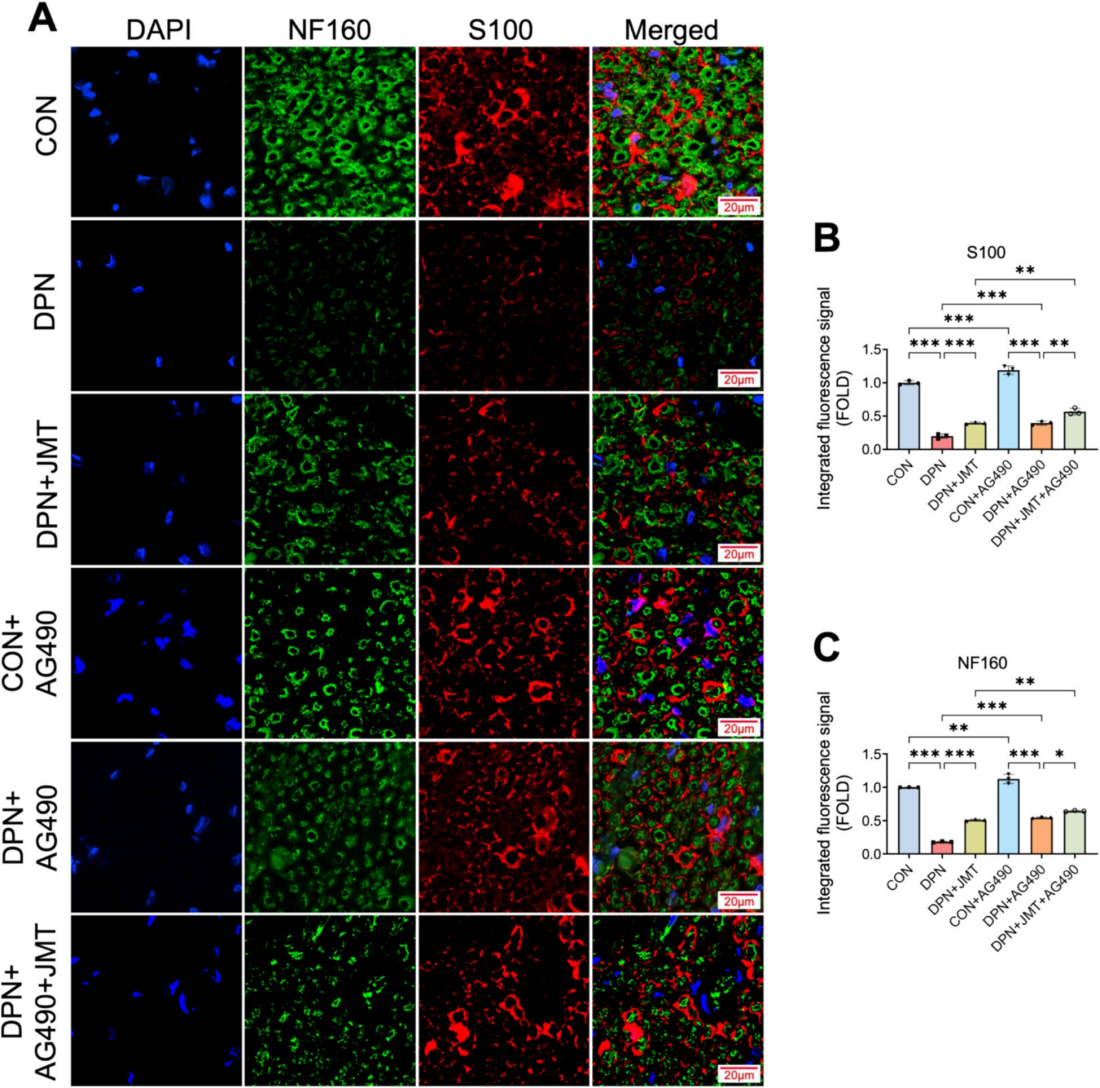
The effect of JMT and AG490 on NF160 and S100 calcium-binding protein in the sciatic nerve of DPN rats. **A** The representative images of immunofluorescence staining of S100 and NF160 in the sciatic nerve (scale bar = 20 µm). **B**, **C** Semi-quantitative analysis of S100 and NF160 immunofluorescence. Data were shown as mean ± SD (n = 3). *P < 0.05, **P < 0.01, ***P < 0.001, *ns*  not significant

**Fig. 9:**
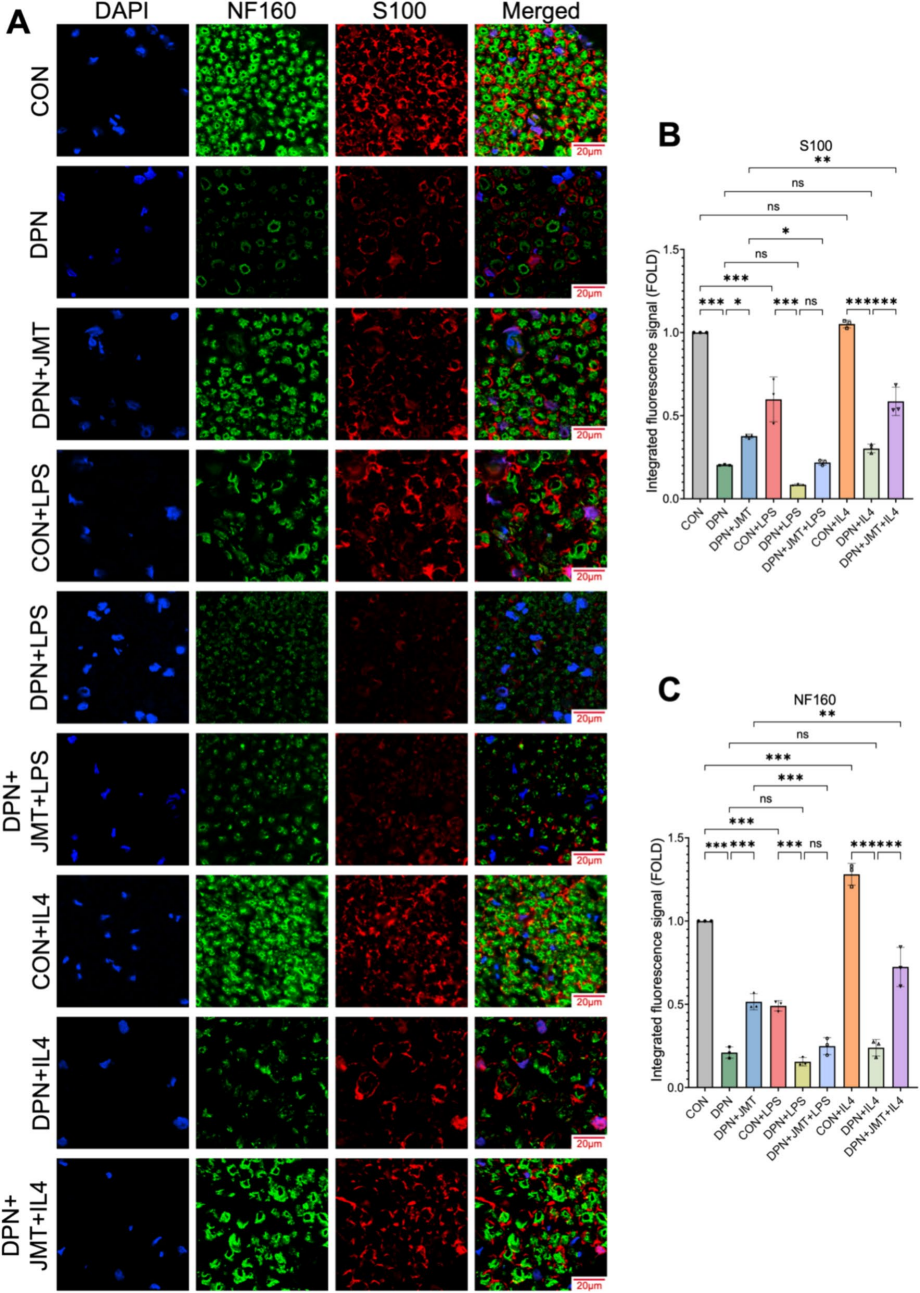
The effect of JMT, LPS, and IL-4 on S100 and NF160 calcium-binding protein in the sciatic nerve of DPN rats. **A** The representative images of immunofluorescence staining of S100 and NF160 in the sciatic nerve (scale bar = 20 µm). **B**, **C** Semi-quantitative analysis of S100 and NF160 immunofluorescence. Data were shown as mean ± SD (n = 3). *P < 0.05, **P < 0.01, ***P < 0.001, *ns*  not significant

Immunofluorescence analysis revealed that the expression levels of the nerve regeneration-associated proteins S100 and NF160 were significantly lower in DPN rats compared to healthy controls (Figs. 8 and 9). Treatment with JMT and AG490 markedly upregulated both proteins, with AG490 showing a synergistic effect when combined with JMT (Fig. 8A–C). Moreover, IL-4 significantly potentiated the JMT-induced upregulation of NF160, whereas LPS attenuated this enhancement. Regarding S100, although the changes did not reach statistical significance, IL-4 exhibited a trend towards increased expression, while LPS showed a tendency to diminish it (Fig. 9A–C).

### JMT promotes macrophage polarization via inhibition of the JAK2/STAT3 pathway

**Fig. 10:**
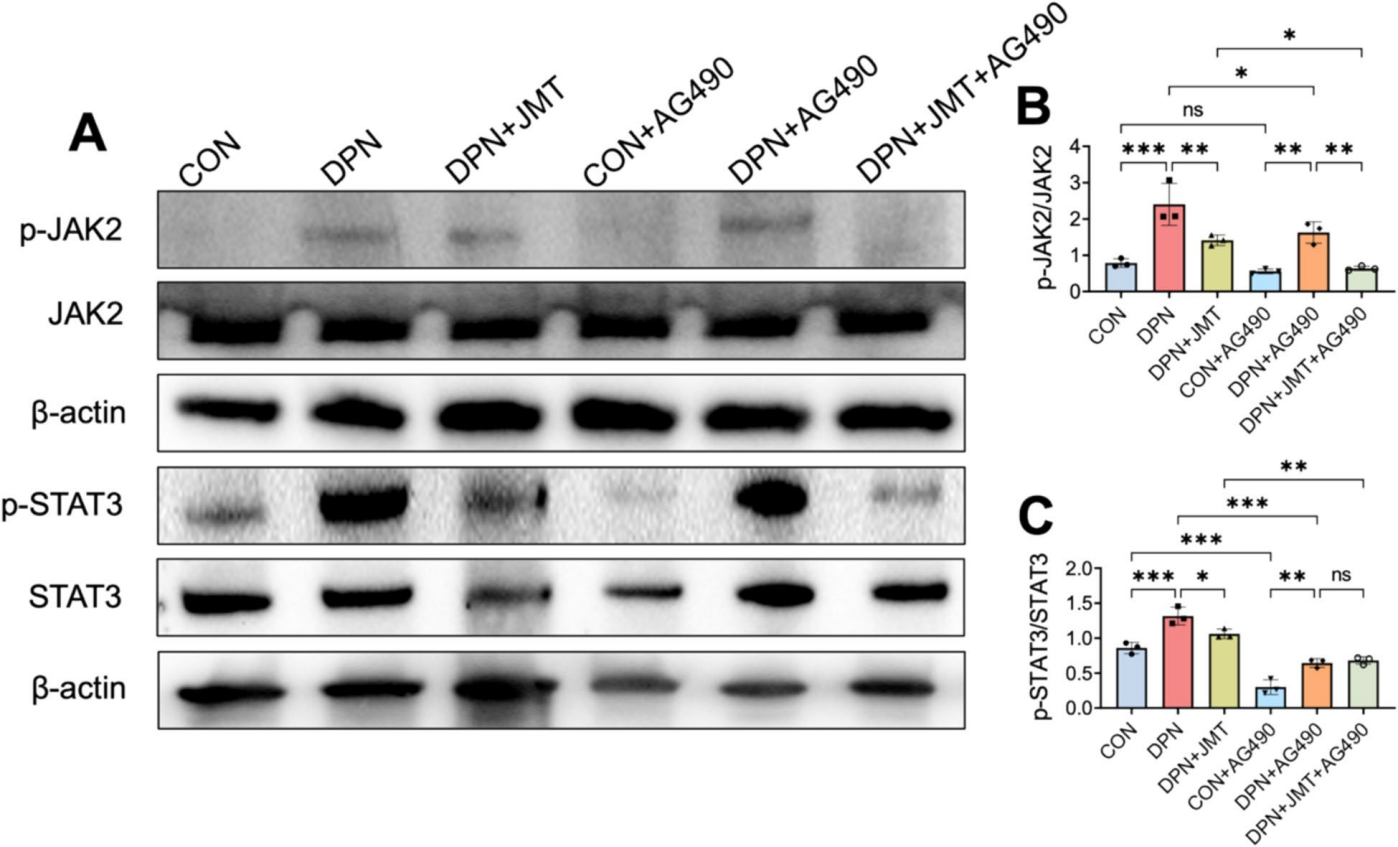
The expression of JAK2/STAT3 signaling related proteins in the sciatic nerves of rats. **A** Representative immunoblots of p-JAK2, JAK2, p-STAT3, and STAT3 in the sciatic nerve. **B**, **C** The analysis of p-JAK2 to JAK2 and p-STAT3 to STAT3. Data were shown as mean ± SD (n = 3). *P < 0.05, **P < 0.01, ***P < 0.001, *ns*  not significant

WB analysis was conducted to evaluate the effect of JMT on the expression of JAK2-STAT3 signaling pathway-related proteins. As shown in Fig. 10A–C, the ratios of p-JAK2/JAK2 and p-STAT3/STAT3 were significantly increased in the sciatic nerves of DPN rats compared to the control group. Notably, treatment with the JAK2 inhibitor AG490 significantly reduced the ratios of p-JAK2/JAK2 and p-STAT3/STAT3. Similarly, JMT treatment alone also significantly reduced the ratios of p-JAK2/JAK2 and p-STAT3/STAT3, and this inhibitory effect was further enhanced when JMT was combined with AG490 (Fig. 10A–C). These findings indicate that JMT exerts an inhibitory effect on the JAK2/STAT3 signaling pathway comparable to that of AG490. In conclusion, these results demonstrate that the JAK2/STAT3 pathway is aberrantly activated in DPN rats, and JMT effectively suppresses this abnormal activation.

### JMT facilitates macrophage polarization and axon regeneration via the JAK2/STAT3 pathway

**Fig. 11:**
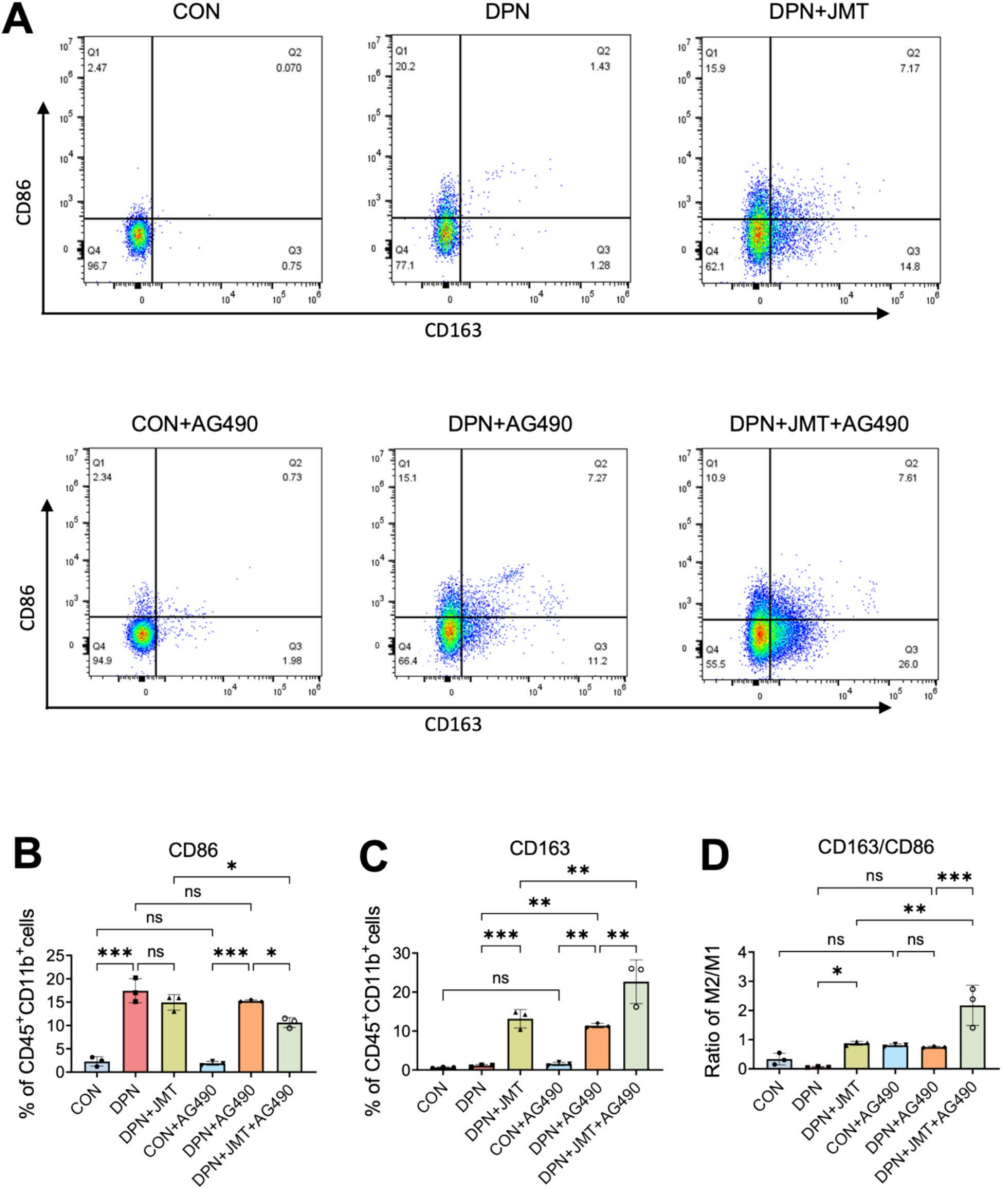
JMT promoted macrophage polarization in the bone marrow of DPN rats. **A**–**D** Flow cytometry was used to analyze single-cell suspensions prepared from rat bone marrow, with fluorescence antibodies CD45 and CD11b used to isolate macrophages, assessing the percentage of M1 macrophages (CD86 +) (**B**) and M2 macrophages (CD163 +) (**C**), and calculating the M2/M1 ratio (**D**). Data were shown as mean ± SD (n = 3). *P < 0.05, **P < 0.01, ***P < 0.001, *ns*  not significant

The JAK2/STAT3 pathway is critically involved in the regulation of macrophage polarization. To elucidate the role of JMT in modulating this pathway and its impact on macrophage phenotypic transformation, flow cytometry was employed to analyze M1 and M2 macrophage populations (Fig. 11A–C). In comparison to the control group, rats with DPN exhibited a marked elevation in the proportion of M1 macrophages (labeled by CD86) in bone marrow-derived macrophages (Fig. 11A, B). Notably, JMT treatment significantly increased the population of M2 macrophages (labeled by CD163) and elevated the M2/M1 macrophage ratio (Fig. 11C, D). A similar trend was observed following treatment with AG490, a known JAK2 inhibitor, and the combination of JMT and AG490 exerted a synergistic effect. These findings indicate that JMT promotes the polarization of macrophages toward the anti-inflammatory M2 phenotype, potentially through suppression of the JAK2/STAT3 signaling cascade, which may contribute to enhanced axonal regeneration in DPN.

**Fig. 12:**
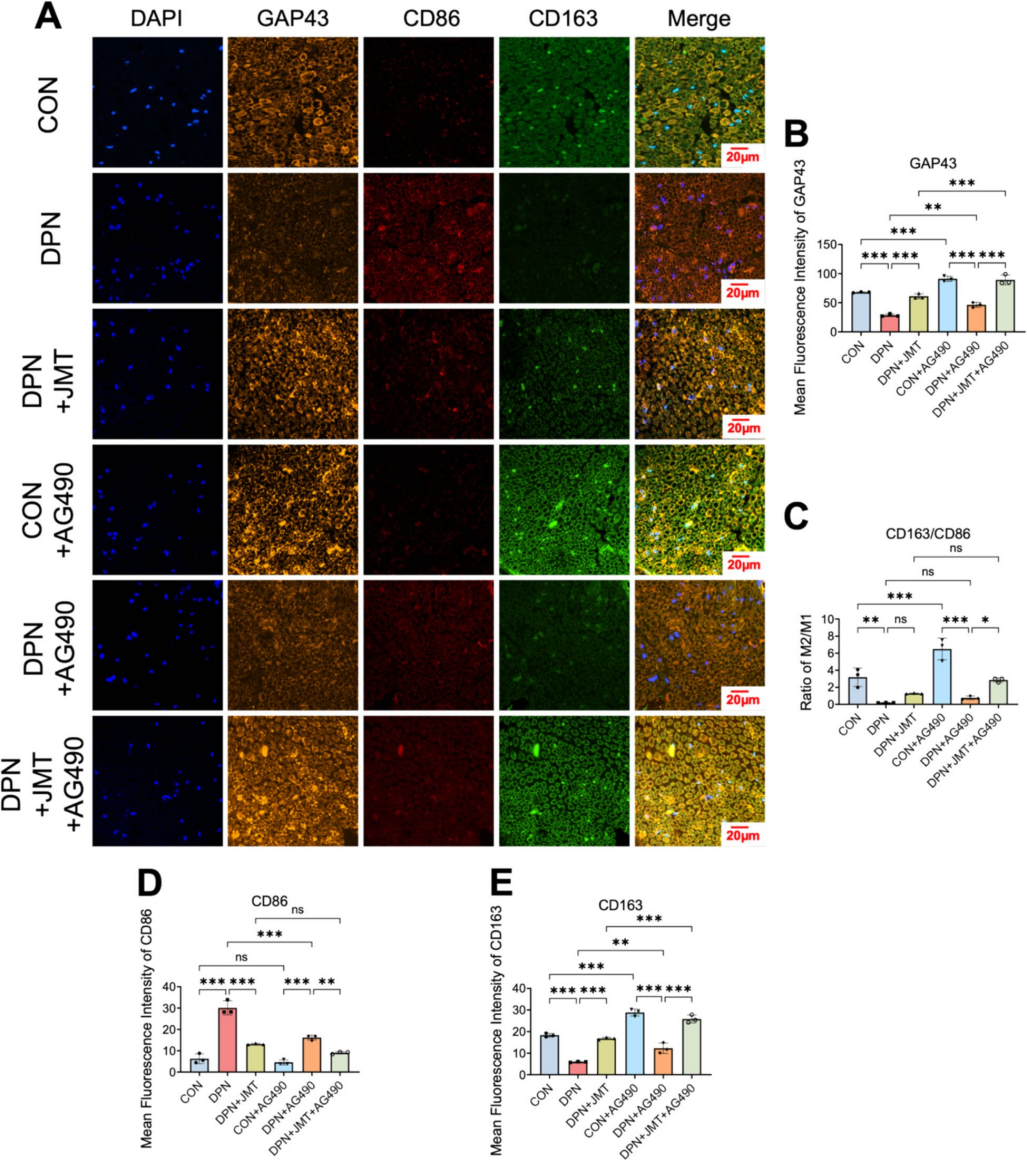
JMT promoted macrophage polarization in the sciatic nerve of DPN rats. **A**–**E **Representative immunofluorescence images and analysis of GAP43 (**B**), M1 macrophages (CD86) (**D**), M2 macrophages (CD163) (**E**) in sciatic nerve (scale bar = 20 µm) and the M2/M1 ratio (**C**). Data were shown as mean ± SD (n = 3). *P < 0.05, **P < 0.01, ***P < 0.001, *ns*  not significant

Furthermore, the polarization status of macrophages (M1 and M2) and nascent axonal regeneration (labeled by GAP43) in the sciatic nerve were examined using immunofluorescence analysis. As shown in Fig. 12A–E, the DPN group exhibited a significant increase in M1 macrophages and a decrease in M2 macrophages compared to the control group. These alterations were reversed by treatment with JMT or AG490. Although the difference was not statistically significant, JMT treatment demonstrated a trend toward promoting the transition from the M1 to the M2 phenotype, and this effect appeared to be further enhanced when combined with AG490. GAP43, a well-established marker of nascent axons and axonal regeneration [44], was markedly reduced in the sciatic nerve of DPN rats (Fig. 12A–B). Remarkably, both JMT and AG490 significantly promoted axonal outgrowth, with JMT exhibiting superior efficacy. Furthermore, co-administration of JMT and AG490 exerted a synergistic effect, further enhancing axonal regeneration. These findings suggest that JMT may facilitate axonal regrowth by promoting M2 macrophage polarization through inhibition of the JAK2/STAT3 signaling pathway.

### JMT mitigates sciatic nerve inflammation in DPN via modulation of the JAK2/STAT3 signaling pathway

**Fig. 13:**
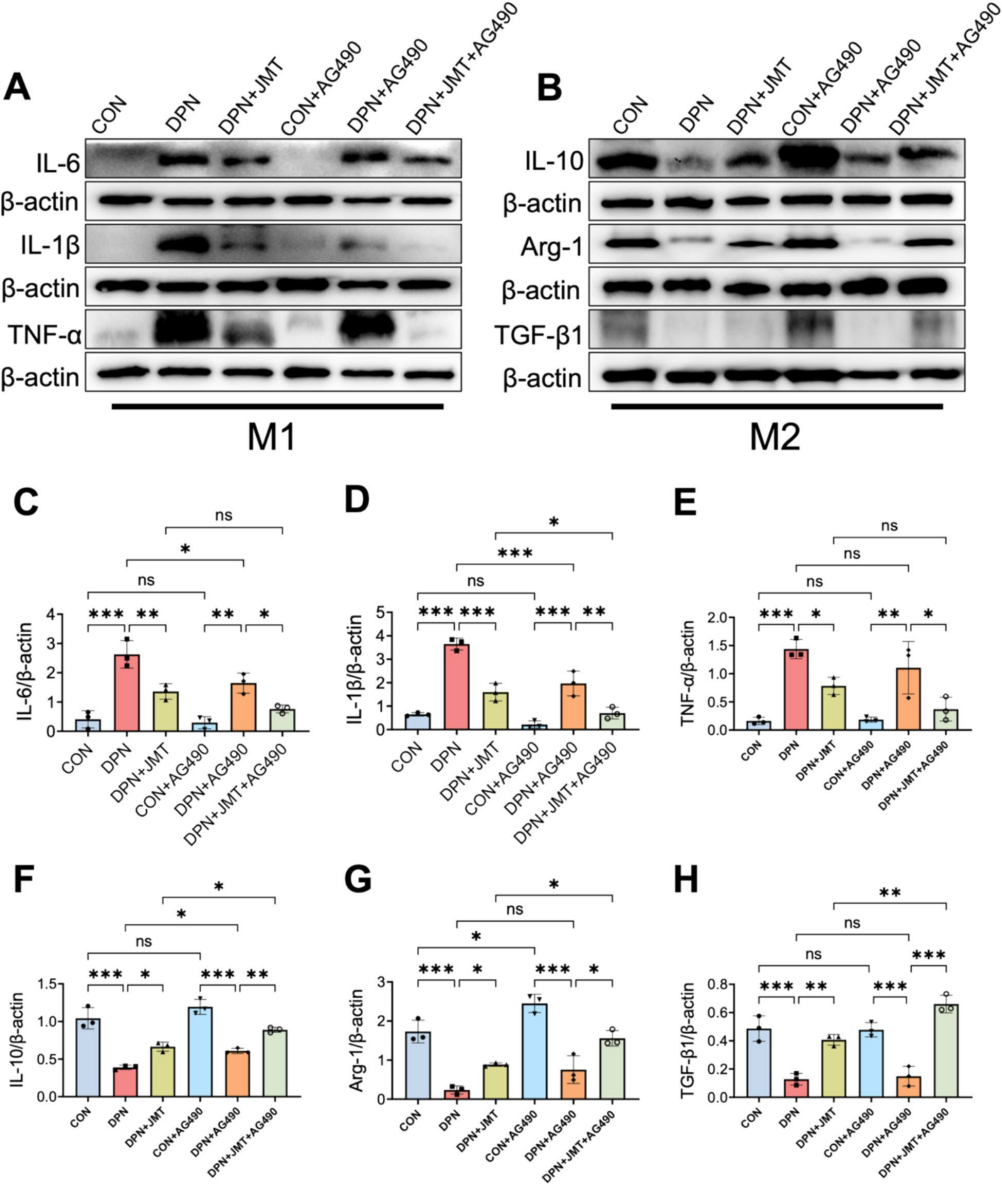
Expression of macrophage polarization-related proteins in the sciatic nerves of rats in each group. **A**–**H** Western blot analysis of macrophage polarization markers, including (**A**) M1 macrophages (pro-inflammatory) markers IL-6 (**C**), IL-1β (**D**), TNF-α (**E**), and (**B)** M2 macrophages (anti-inflammatory) markers IL-10 (**F**), Arg-1 (**G**), and TGF-β1 (**H**). Data were shown as mean ± SD (n = 3). *P < 0.05, **P < 0.01, ***P < 0.001, *ns*  not significant

Macrophages typically exhibit polarization into two distinct phenotypes: M1 and M2. The M1 phenotype, upon activation, contributes to neuroinflammatory processes by producing high levels of pro-inflammatory cytokines, including IL-1β, IL-6, and TNF-α. Conversely, M2 macrophages are associated with the expression of anti-inflammatory mediators such as IL-10, Arg-1, and TGF-β1 [45]. To further investigate neuroinflammatory alterations, levels of inflammatory cytokines in the sciatic nerves of DPN rats were analyzed via Western blot. As illustrated in Fig. 13A–H, DPN rats exhibited significantly increased expression of IL-6, IL-1β, and TNF-α, alongside a notable reduction in anti-inflammatory cytokines IL-10, Arg-1, and TGF-β1.

Treatment with JMT markedly suppressed the expression of pro-inflammatory cytokines while concurrently upregulating the levels of anti-inflammatory mediators, indicating its potent immunomodulatory effect. Similarly, administration of AG490 significantly reduced the expression of IL-6 and IL-1β, and notably increased IL-10 levels. Although reductions in TNF-α and elevations in Arg-1 and TGF-β1 were observed, these changes did not reach statistical significance (Fig. 13A–H). Furthermore, combined treatment with JMT and AG490 exhibited a synergistic effect, leading to a more pronounced decrease in pro-inflammatory cytokine levels and a significant enhancement in the expression of anti-inflammatory markers (Fig. 13A–H).

## Discussion

DPN is highly prevalent among diabetic patients and poses a significant threat to human health [46]. Despite numerous clinical interventions, therapeutic outcomes remain unsatisfactory [47]. Traditional Chinese medicine, such as JMT, has emerged as a promising alternative, exhibiting both favorable clinical efficacy and safety [17, 18]. Our previous studies demonstrated that JMT improved peripheral neurological function in T1DM rats [20, 48]. Given that the majority of DPN cases occur in patients with T2DM [49], the present study aimed to investigate the therapeutic potential of JMT in T2DM-induced DPN.

Based on the safe and effective doses verified in preliminary experiments by our research team, we selected the following JMT administration doses for this study: a low dose equivalent to the clinical dose (7.6 mg/kg) (equal to clinical dose, based on the weight of JMT lyophilized powder) and a high dose (15.2 mg/kg) [24]. Our results revealed that JMT significantly improved peripheral nerve function in T2DM rats, as evidenced by increased MNCV, elevated mechanical thresholds, reduced thermal latency, and mitigation of demyelination and axonal damage. Notably, the tail-flick test was more sensitive than the hot-plate test in assessing thermal nociception. The observed improvements were both dose- and time-dependent, with high-dose JMT showing the most pronounced neuroprotective effects after 12 weeks of treatment. Importantly, JMT did not influence blood glucose levels or body weight, suggesting its therapeutic effects were independent of glycemic control.

Accumulating evidence implicates chronic low-grade inflammation in the pathogenesis of DPN [50]. Transcriptomic analyses of sciatic nerve tissue from DPN models have identified significant dysregulation in immune and inflammatory signaling pathways [51]. At the protein level, elevated levels of IL-6, IL-1β, and TNF-α correlate with reduced MNCV and increased neuropathic pain [52]. Our data support these findings, showing increased expression of pro-inflammatory mediators (iNOS, MCP-1, IL-1β) and reduced levels of anti-inflammatory cytokines (IL-10, TGF-β1) in both serum and sciatic nerve from week eight onward. Consistent with prior studies indicating the protective role of IL-4 and IL-10 in DPN [53], our results showed that high-dose JMT upregulated IL-10 and TGF-β1 while suppressing pro-inflammatory cytokines. These effects suggest JMT exerts potent anti-inflammatory and neuroprotective effects.

Macrophage polarization plays a central role in regulating inflammatory responses in peripheral nerve injury. Our gene ontology analysis indicated that JMT modulates macrophage colony-stimulating factor receptor activity, implicating its role in macrophage-driven neuroinflammation. Classically activated M1 macrophages release pro-inflammatory factors such as IL-1β and TNF-α, while alternatively activated M2 macrophages produce anti-inflammatory mediators like IL-10 and Arg-1 [8, 54]. Macrophage polarization is pivotal in nerve repair and regeneration [55]. In our second-phase experiments, the beneficial effects of JMT were validated through interventions using the M1 activator LPS and the M2 activator IL-4. While JMT and IL-4 significantly improved nerve function and histopathology, LPS exacerbated neuropathy. Furthermore, JMT significantly upregulated NF160 and S100 expression—markers of axonal regeneration and Schwann cells, respectively. Co-treatment with IL-4 enhanced JMT’s effect, while LPS diminished it, suggesting macrophage polarization is a critical mediator of JMT’s therapeutic action.

Additionally, UPLC-HRMS analysis further identified the main components in JMT, including 4-coumaric acid, aurantio-obtusin-glucoside, formononetin, quercetin, loliolide, and others. Based on PPI network analysis, the core targets of JMT intervention in DPN (such as TNF, IL2, TLR4, STAT3, JAK2, AKT1, RELA, PTGS2, TGFB1, CCND1) were all closely associated with macrophage functions. Network pharmacology enrichment analysis revealed that these targets were significantly enriched in inflammation-related regulatory pathways including TNF, IL-17, and JAK/STAT signaling. The molecular docking results also demonstrate the strong binding affinity of JMT’s top six primary core components—4-Coumaric acid, Formononetin, Quercetin, Loliolide, L-phenylalanine, and Caffeic acid—screened through the SWISSADME database, with both JAK2 and STAT3. Notably, the JAK/STAT pathway, serving as a canonical upstream regulatory mechanism for macrophage polarization, may mediate JMT’s immunomodulatory effects through activation of JAK2 and STAT3 (key hubs in the PPI network), thereby altering the balance between pro-inflammatory (M1) and anti-inflammatory (M2) phenotypes.

In the first experimental phase, we first observed significant cytokine profile changes at week eight. Therefore, we initiated AG490 treatment via intraperitoneal injection starting from this time point to investigate the JAK2/STAT3 pathway’s role. This design aimed to explore the regulatory role of the JAK2/STAT3 pathway in JMT-mediated macrophage polarization and neuroprotection. Experimental results indicated no significant differences in behavioral tests and immunofluorescence findings for S100 and NF160 between diabetic rats injected with LPS and those without LPS. We speculate that this may be due to the nervous system in the diabetic condition having reached a ‘saturation phase’ of inflammatory damage, where the extent of functional impairment is near its maximum under the current pathological conditions, rendering it unresponsive to further inflammatory stimuli. Future research will continue to further validate this hypothesis.

The JAK2/STAT3 signaling pathway, which plays a vital role in macrophage polarization and inflammatory regulation, was further explored in this study [43]. JAK2 activation leads to STAT3 phosphorylation and nuclear translocation, regulating downstream gene expression [56]. This pathway has been shown to be activated following nerve injury and in high-glucose conditions [57, 58]. In our study, inhibition of JAK2 using AG490 mimicked the beneficial effects of IL-4, enhancing JMT’s neuroprotective outcomes and reversing LPS-induced damage. Moreover, both JMT and AG490 downregulated the phosphorylation of JAK2 and STAT3 in sciatic nerves, with their combination yielding an additive effect. However, it must be prudently noted that cellular signaling networks are typically dynamic and highly interconnected, with the possibility of bidirectional regulation. A substantial body of literature reports that typical M2-type cytokines (such as IL-10) can feedback through their receptors to activate STAT3, forming a positive feedback loop to stabilize the M2 phenotype. This complex signal crosstalk implies that the role of the JAK2-STAT3 pathway may not be static across different stages of macrophage polarization or under varying pathological contexts, although our data primarily support its function as an upstream driving event. This intricate temporal dynamics and network interplay will be an important direction for future research, for instance, by employing time-series experiments to dynamically track the sequence of JAK2/STAT3 phosphorylation and the expression of M2 markers.

To further confirm the involvement of the JAK2/STAT3 axis in macrophage polarization, flow cytometry analysis of bone marrow-derived macrophages showed that JMT increased M2 polarization and the M2/M1 ratio in DPN rats. These effects were potentiated by AG490. Similarly, in the sciatic nerve, JMT reduced M1 and elevated M2 macrophage populations, effects enhanced by AG490 co-treatment. JMT also upregulated GAP43, a marker of axonal regeneration. Co-staining revealed a correlation between increased M1 macrophages and reduced GAP43 expression, and vice versa for M2 macrophages. These findings strongly suggest that JMT promotes axonal regeneration through JAK2/STAT3-mediated macrophage polarization. Of particular note, there are currently no drugs specifically approved for the treatment of DPN. Our research seeks to advance potential treatment options for this condition. AG490 and IL-4 served as positive controls in this experiment, given their established efficacy in DPN [59–61].

In summary, JMT exerts neuroprotective effects in T2DM-induced DPN by modulating macrophage polarization and suppressing inflammation via inhibition of the JAK2/STAT3 signaling pathway. These effects promote axonal regeneration and remyelination, thereby alleviating peripheral neuropathy. The findings from the second experimental phase further substantiate JMT’s ability to suppress LPS-induced M1 polarization and enhance IL-4-mediated M2 transition. While the study provides robust evidence supporting the therapeutic potential of JMT, limitations remain. The molecular crosstalk between JMT and the JAK2/STAT3 pathway warrants deeper mechanistic exploration. We first characterized the chemical profile of JMT using liquid chromatography coupled with high-resolution mass spectrometry. Subsequently, we employed network pharmacology and molecular docking to predict the signaling pathways targeted by JMT in DPN treatment. The disease targets for DPN were obtained from public databases, and the active components of JMT against DPN were identified through computational simulations. Nonetheless, it is important to acknowledge that these approaches are inherently predictive, reflecting a common limitation of network pharmacology. Despite this, our findings establish a valuable theoretical framework and propose scientific hypotheses for understanding the pharmacological mechanisms of JMT. In the near future, we would focus on integrating multidisciplinary technologies, such as thermal proteome profiling [62], to further validate and explore these mechanisms in greater depth.

## Conclusions

In conclusion, this study demonstrated that high-dose administration of JMT for eight weeks significantly alleviated DPN in a T2DM rat model. The neuroprotective effects were evidenced by improved motor nerve conduction velocity, reduced mechanical and thermal pain sensitivity, and alleviation of axonal and myelin damage.

Importantly, JMT was shown to promote a phenotypic switch in macrophages from the pro-inflammatory M1 type to the anti-inflammatory M2 type, thereby reducing the expression of pro-inflammatory cytokines (e.g., IL-1β, iNOS, MCP-1) and increasing the expression of anti-inflammatory mediators (e.g., IL-10, TGF-β1). This immunomodulatory effect was found to be mediated, at least in part, through the inhibition of the JAK2/STAT3 signaling pathway, as demonstrated by pharmacological intervention using AG490 and subsequent changes in macrophage polarization and neuroinflammation.

These findings not only elucidate the cellular and molecular mechanisms underlying JMT’s therapeutic efficacy in DPN but also suggest that targeting macrophage polarization via JAK2/STAT3 signaling could be a promising strategy for future intervention. Further investigation is warranted to dissect the detailed interactions between the bioactive components of JMT and key inflammatory signaling pathways, and to validate these results in clinical settings.

## Supplementary Information

Supplementary material 1.

## Data Availability

No datasets were generated or analysed during the current study.

## Abbreviations

ANOVA: Analysis of variance
Arg-1: Arginase-1
BC: Betweenness centrality
BCA: Bicinchoninic acid assay
CC: Closeness centrality
cDNA: Complementary DNA
DC: Degree centrality
DM: Diabetes mellitus
DPN: Diabetic peripheral neuropathy
ELISA: Enzyme-linked immunosorbent assay
ESI: Electrospray ionization
GAP43: Growth associated protein 43
GO: Gene ontology
HFD: High fat diet
HRP: Horseradish peroxidase
H&E: Hematoxylin and Eosin
iNOS: Inducible nitric oxide synthase
IL-1β: Interleukin-1 beta
IL-4: Interleukin-4
IL-6: Interleukin-6
IL-10: Interleukin-10
IL-18: Interleukin-18
JAK2: Janus kinase 2
JMT: Jinmaitong
KEGG: Kyoto encyclopedia of genes and genomes
LC-MS: Liquid chromatography-mass spectrometry
LFB: Luxol fast blue
LPS: Lipopolysaccharide
MCP-1: Monocyte chemoattractant protein-1
MNCV: Motor nerve conduction velocity
NF160: Neurofilament 160
OD: Optical density
PBS: Phosphate-buffered saline
PCR: Polymerase chain reaction
PFA: Paraformaldehyde
pH: Potential of hydrogen
PPI: Protein-protein interaction
PVDF: Polyvinylidene fluoride
qPCR: Quantitative polymerase chain reaction
RIPA: Radioimmunoprecipitation assay buffer
SD: Sprague-Dawley
SPF: Specific pathogen-free
STAT3: Signal transducer and activator of transcription 3
STZ: Streptozotocin
TBST: Tris-buffered saline with Tween
TEM: Transmission electron microscopy
TGF-β1: Transforming growth factor-beta 1
TICs: Total ion chromatograms
TNF-α: Tumor necrosis factor alpha
T1DM: Type 1 diabetes
T2DM: Type 2 diabetes mellitus
UPLC-HRMS: Ultra-performance liquid chromatography coupled with high-resolution mass spectrometry
