# Supplementary material for: Elucidation of the mechanism of Jinmaitong against Diabetic peripheral neuropathy based on a combined strategy of network pharmacology and molecular biology
Source: Chin Med. 2026 Feb 2;21:60. doi: 10.1186/s13020-025-01300-0 (PMC12866583; doi:10.1186/s13020-025-01300-0)
Supplement: Supplementary file 1 — Supplementary material 1. [file 13020_2025_1300_MOESM1_ESM.docx]

**Table S1.** The origin of the crude drugs in Jinmaitong (JMT) decoction

| Drug name | Botanical origins | Chinese name | Ratio |
| --- | --- | --- | --- |
| Semen Cuscutae | the seeds of *Cuscuta chinensis* Lam. | Tu-Si-Zi | 10 |
| Ramulus Cinnamomi | the tender stem of *Cinnamomum cassia* Presl. | Gui-Zhi | 10 |
| Herba Ecliptae | the herb of *Eclipta prostrata* L. | Mo-Han-Lian | 10 |
| Herba Prunella Vulgaris | the herb of *Prunella vulgaris* L. | Xia-Ku-Cao | 10 |
| Fructus Ligustri Lucidi | the seeds of *Ligustrum lucidum* Ait. | Nv-Zhen-Zi | 10 |
| Scorpio | *Buthus martensii* K. | Quan-Xie | 3 |
| Semen Litchi | the seeds of *Litchi chinensis* Sonn. | Li-Zhi-He | 30 |
| Rhizoma Corydalis | the rhizoma of *Corydalis yanhusuo* W. T. Wang | Yan-Hu-Suo | 10 |
| Semen Persicae | the seeds of *Prunus persica* L. | Tao-Ren | 10 |
| Radix et Rhizoma Asari | the radix and rhizoma of *Asarum heterotropiodes* F. | Xi-Xin | 3 |
| Hirudo | *Hirudo nipponica* W. | Shui-Zhi | 3 |
| Semen Cassiae | the seeds of *Cassia obtusifolia* L. | Jue-Ming-Zi | 30 |

**Table S2.** Drugs, chemicals, and reagents

| Name | Manufacturer | Country | Cat No. |
| --- | --- | --- | --- |
| STZ | Sigma-Aldrich | USA | 572201 |
| AG490 | TargetMol | USA | T2600 |
| Recombinant Rat IL4 | Beyotime | China | P6267 |
| LPS | Beyotime | China | S1732 |
| Rat TGF-β1(Transforming Growth Factor Beta 1) ELISA Kit | Elabscience | China | E-EL-0162 |
| Rat NOS2/iNOS(Nitric Oxide Synthase 2, Inducible) ELISA Kit | Elabscience | China | E-EL-R0520c |
| Rat IL-10(Interleukin 10) ELISA Kit | Elabscience | China | E-EL-R0016c |
| Rat IL-1β(Interleukin 1 Beta) ELISA Kit | Elabscience | China | E-EL-R0012c |
| Rat MCP-1(Monocyte Chemotactic Protein 1) ELISA Kit | Elabscience | China | E-EL-R0633c |
| Rat TNF-α(Tumor Necrosis Factor Alpha) ELISA Kit | Elabscience | China | E-EL-R2856c |
| Anti-CD163/PE | Bioss | China | bs-2527R-PE |
| Zombie Aqua^TM^ Fixable Viability Kit | Biolegend | USA | 423101 |
| Anti-Rat CD45 OX1 APC-EF780 | ThermoFisher | USA | 47-0461-82 |
| APC anti-rat CD11b/c Antibody | Biolegend | USA | 201809 |
| Anti-Rat CD86 24F FITC | ThermoFisher | USA | 11-0860-82 |
| RNA isolater Total RNA Extraction Reagent | Vazyme | China | R401-01 |
| HiScript III RT SuperMix for qPCR（+gDNA wiper） | Vazyme | China | R333-01 |
| HiScript IV All-in-One Ultra RT SuperMix for qPCR | Vazyme | China | R433-01 |
| JAK2 Antibody | Affinity | China | AF6022 |
| Phospho-JAK2 (Tyr931) Antibody | Affinity | China | AF3024 |
| TNF alpha Antibody | Affinity | China | AF7014 |
| IL6 Antibody | Affinity | China | DF6087 |
| IL10 Antibody | Affinity | China | DF6894 |
| ARG1 Antibody | Affinity | China | DF6657 |
| Phospho-STAT3 (Tyr705) Antibody | Affinity | China | AF3293 |
| STAT3 Antibody | Affinity | China | AF6294 |
| IL1 beta Antibody | Affinity | China | AF5103 |
| TGF beta 1 Antibody | Affinity | China | AF1027 |
| β-Actin Mouse mAb | ABclonal | China | AC004 |
| Rabbit Anti-S100B antibody | Bioss | China | bs-2015R |
| GAP43 Polyclonal Antibody | ThermoFisher | USA | PA5-95660 |
| Anti-160 kD Neurofilament Medium | Abcam | UK | ab7794 |
| Rabbit Anti-CD86 antibody | Bioss | China | bs-2527R |
| CD163 Monoclonal Antibody (ED2) | ThermoFisher | USA | MA5-16656 |
| CoraLite488-conjugated Goat Anti-Mouse IgG(H+L) | Proteintech | China | SA00013-1 |
| CoraLite594-conjugated Goat Anti-Rabbit IgG(H+L) | Proteintech | China | SA00013-4 |
| Goat anti-Chicken IgY (H+L) Secondary Antibody, Alexa Fluor™ 647 | ThermoFisher | USA | A-21449 |
| Rat ACTB Endogenous Reference Genes Primer | Sangon Biotech | China | B661202 |

**Table S3.** Primer sequences

| **Gene name** | **Forward primer (5′-3′)** | **Reverse primer (5′-3′)** |
| --- | --- | --- |
| MCP-1 | CACCTGCTGCTACTCATTCACTGG | CTTCTTTGGGACACCTGCTGCTG |
| IL-1β | CTCACAGCAGCATCTCGACAAGAG | TCCACGGGCAAGACATAGGTAGC |
| iNOS | ACCGAGATTGGAGTCCGAGA | GCACAGCTGCATTGATCTCG |
| TGF-β1 | GACCGCAACAACGCAATCTATGAC | CTGGCACTGCTTCCCGAATGTC |
| IL-10 | CGCATCCAGACACACACAGACTAG | GCCCAGAGACAGACAAGCAAGAG |

## Table S4. Composition of Jinmaitong

| Metabolite | m/z | Retention time | Formula | Fragmentation Score | Classification | SMILES | Whether passed the SwissADEM screening |
| --- | --- | --- | --- | --- | --- | --- | --- |
| 4-Coumaric acid | 147.0440611 | 2.931533333 | C9H8O3 | 97.9 | Phenolic acids and derivatives | O=C(O)C=CC1=CC=C(O)C=C1 | YES |
| Aurantio-obtusin beta-D-glucoside | 493.1336086 | 5.024833333 | C23H24O12 | 98 | - | COC1=C(OC2OC(CO)C(O)C(O)C2O)C=C2C(=O)C3=C(C(=O)C2=C1O)C(OC)=C(O)C(C)=C3 | NO |
| Formononetin | 269.0806408 | 8.4761 | C16H12O4 | 96.5 | Flavonoids | COC1=CC=C(C2=COC3=C(C=CC(O)=C3)C2=O)C=C1 | YES |
| Quercetin | 303.0496982 | 4.072016667 | C15H10O7 | 98.3 | Flavonoids | O=C1C(O)=C(C2=CC(O)=C(O)C=C2)OC2=CC(O)=CC(O)=C12 | YES |
| Loliolide | 197.1172391 | 4.544616667 | C11H16O3 | 95.8 | Others | CC1(C)CC(O)CC2(C)OC(=O)C=C12 | YES |
| L-phenylalanine | 166.0862787 | 1.971466667 | C9H11NO2 | 95 | Amino acids and derivatives | NC(CC1=CC=CC=C1)C(=O)O | YES |
| Nuezhenide | 709.2306204 | 4.437216667 | C31H42O17 | 98.7 | Lipids | CC=C1C(OC2OC(CO)C(O)C(O)C2O)OC=C(C(=O)OC)C1CC(=O)OCC1OC(OCCC2=CC=C(O)C=C2)C(O)C(O)C1O | NO |
| Caffeic acid | 179.0344408 | 3.270816667 | C9H8O4 | 96.4 | Phenolic acids and derivatives | O=C(O)C=CC1=CC(O)=C(O)C=C1 | YES |
| Rubrofusarin | 273.0754403 | 4.9836 | C15H12O5 | 96.2 | Others | COC1=CC(O)=C2C(O)=C3C(=O)C=C(C)OC3=CC2=C1 | YES |
| Amygdalin | 458.165291 | 3.277383333 | C20H27NO11 | 96.7 | Carbohydrates and derivatives | N#CC(OC1OC(COC2OC(CO)C(O)C(O)C2O)C(O)C(O)C1O)C1=CC=CC=C1 | NO |
| Aurantio-obtusin | 331.0808641 | 5.024833333 | C17H14O7 | 97.6 | Quinones | COC1=C(O)C=C2C(=O)C3=C(C(=O)C2=C1O)C(OC)=C(O)C(C)=C3 | YES |
| Isoquercetin | 465.1025545 | 4.072016667 | C21H20O12 | 94 | Flavonoids | O=C1C(OC2OC(CO)C(O)C(O)C2O)=C(C2=CC(O)=C(O)C=C2)OC2=CC(O)=CC(O)=C12 | NO |
| Morin | 303.0497166 | 3.8627 | C15H10O7 | 94.8 | Flavonoids | O=C1C(O)=C(C2=C(O)C=C(O)C=C2)OC2=CC(O)=CC(O)=C12 | YES |
| Coptisine | 320.0914984 | 4.881683333 | C19H14NO4+ | 95.9 | Alkaloids and derivatives | C1=C2CC[N+]3=C(C=C4C=CC5=C(OCO5)C4=C3)C2=CC2=C1OCO2 | YES |
| 2-methoxycinnamaldehyde | 163.0753154 | 8.042533333 | C10H10O2 | 91.3 | Others | COC1=CC=CC=C1C=CC=O | YES |
| Cynaroside | 449.1073543 | 4.1999 | C21H20O11 | 98.8 | Flavonoids | O=C1C=C(C2=CC(O)=C(O)C=C2)OC2=CC(OC3OC(CO)C(O)C(O)C3O)=CC(O)=C12 | NO |
| Oleamide | 282.2789208 | 13.70431667 | C18H35NO | 93.5 | Lipids | CCCCCCCCC=CCCCCCCCC(N)=O | YES |
| 3,5-Di-O-caffeoylquinic acid | 515.1201967 | 4.647233333 | C25H24O12 | 94.3 | Phenolic acids and derivatives | O=C(C=CC1=CC(O)=C(O)C=C1)OC1CC(O)(C(=O)O)CC(OC(=O)C=CC2=CC(O)=C(O)C=C2)C1O | NO |
| Obtusifolin | 285.0756072 | 6.682116667 | C16H12O5 | 97.5 | Quinones | COC1=C(O)C(C)=CC2=C1C(=O)C1=C(C=CC=C1O)C2=O | YES |
| Jatrorrhizine | 338.1382889 | 4.69495 | C20H20NO4+ | 93.9 | Alkaloids and derivatives | COC1=C(O)C=C2CC[N+]3=C(C=C4C=CC(OC)=C(OC)C4=C3)C2=C1 | YES |
| Isorhamnetin | 317.0653329 | 4.458583333 | C16H12O7 | 90.5 | Flavonoids | COC1=C(O)C=CC(C2=C(O)C(=O)C3=C(O)C=C(O)C=C3O2)=C1 | YES |
| Abscisic acid | 265.1432287 | 5.75225 | C15H20O4 | 90.5 | Terpenoids | CC(C=CC1(O)C(C)=CC(=O)CC1(C)C)=CC(=O)O | YES |
| Protopine | 354.1333822 | 4.47955 | C20H19NO5 | 89.6 | Alkaloids and derivatives | CN1CCC2=CC3=C(C=C2C(=O)CC2=C(C1)C1=C(C=C2)OCO1)OCO3 | YES |
| Chrysophanol | 255.0650134 | 5.1077 | C15H10O4 | 92.1 | Quinones | CC1=CC2=C(C(=O)C3=C(C=CC=C3O)C2=O)C(O)=C1 | YES |
| Glaucine | 356.185197 | 4.7587 | C21H25NO4 | 90.2 | Alkaloids and derivatives | COC1=C(OC)C=C2C(=C1)CC1C3=C2C(OC)=C(OC)C=C3CCN1C | YES |
| Cryptochlorogenic acid | 353.0882359 | 3.005366667 | C16H18O9 | 89.2 | Phenolic acids and derivatives | O=C(C=CC1=CC(O)=C(O)C=C1)OC1C(O)CC(O)(C(=O)O)CC1O | NO |
| Adenosine | 268.1037362 | 0.646416667 | C10H13N5O4 | 88 | Carbohydrates and derivatives | NC1=C2N=CN(C3OC(CO)C(O)C3O)C2=NC=N1 | NO |
| Palmatine | 352.1538972 | 5.3394 | C21H22NO4+ | 90.5 | Alkaloids and derivatives | COC1=C(OC)C=C2C(=C1)CC[N+]1=C2C=C2C=CC(OC)=C(OC)C2=C1 | YES |
| Dehydrocorydaline | 366.169653 | 5.7745 | C22H24NO4+ | 89.4 | Alkaloids and derivatives | COC1=CC2=CC=[N+]3C=C4C5=C(C=CC4=C(C)C3=C2C=C1OC)OCO5 | YES |
| Astragalin | 447.094021 | 4.191583333 | C21H20O11 | 89.4 | Flavonoids | O=C1C(OC2OC(CO)C(O)C(O)C2O)=C(C2=CC=C(O)C=C2)OC2=CC(O)=CC(O)=C12 | NO |
| Cinnamyl alcohol | 117.070208 | 3.8014 | C9H10O | 87.1 | Others | OCC=CC1=CC=CC=C1 | YES |
| Salidroside | 301.1278774 | 2.749633333 | C14H20O7 | 90.2 | Carbohydrates and derivatives | OCC1OC(OCCC2=CC=C(O)C=C2)C(O)C(O)C1O | YES |
| 2-Hydroxycinnamic acid | 147.0439987 | 5.190083333 | C9H8O3 | 83.7 | Phenolic acids and derivatives | O=C(O)C=CC1=CC=CC=C1O | YES |
| Caffeoyl quinic acid | 355.1020548 | 2.993066667 | C16H18O9 | 89.6 | Phenolic acids and derivatives | O=C(C=CC1=CC(O)=C(O)C=C1)OC1CC(O)(C(=O)O)CC(O)C1O | NO |
| Cinnamic acid | 131.0492667 | 3.923466667 | C9H8O2 | 91.4 | Organic acids and derivatives | O=C(O)C=CC1=CC=CC=C1 | YES |
| Oleuropein | 539.178009 | 5.099116667 | C25H32O13 | 85.5 | Terpenoids | CC=C1C(OC2OC(CO)C(O)C(O)C2O)OC=C(C(=O)OC)C1CC(=O)OCCC1=CC(O)=C(O)C=C1 | NO |
| Choline | 104.1074677 | 0.57465 | C5H14NO+ | 81.3 | Others | C[N+](C)(C)CCO | NO |
| Rosmarinate | 359.0776069 | 4.75955 | C18H16O8 | 79.5 | Phenolic acids and derivatives | O=C(C=CC1=CC=C(O)C(O)=C1)OC(CC1=CC=C(O)C(O)=C1)C(=O)O | NO |
| Cinnamaldehyde | 133.0649124 | 4.156816667 | C9H8O | 77 | Others | O=CC=CC1=CC=CC=C1 | YES |
| Diosmetin | 299.0565083 | 7.922216667 | C16H12O6 | 78.7 | Flavonoids | COC1=C(O)C=C(C2=CC(=O)C3=C(C=C(O)C=C3O)O2)C=C1 | YES |
| Gardenin B | 357.0985105 | 6.3982 | C19H18O7 | 80.5 | Flavonoids | COC1=CC=C(C2=CC(=O)C3=C(O)C(OC)=C(OC)C(OC)=C3O2)C=C1 | YES |
| Alternariol | 259.0600884 | 6.105033333 | C14H10O5 | 75.9 | Coumarins and derivatives | CC1=CC(O)=CC2=C1C1=C(C(O)=CC(O)=C1)C(=O)O2 | YES |
| Glycitein | 283.0615468 | 9.901433333 | C16H12O5 | 81 | Flavonoids | COC1=C(O)C=C2OC=C(C3=CC=C(O)C=C3)C(=O)C2=C1 | YES |
| Aloeemodin | 269.0458932 | 12.15233333 | C15H10O5 | 78.8 | Quinones | O=C1C2=C(C(=O)C3=C1C=C(CO)C=C3O)C(O)=CC=C2 | YES |
| Allocryptopine | 370.1645228 | 4.7162 | C21H23NO5 | 74.3 | Alkaloids and derivatives | COC1=C(OC)C2=C(C=C1)CC(=O)C1=CC3=C(C=C1CCN(C)C2)OCO3 | YES |
| Caryophyllene epoxide | 221.1898038 | 3.986016667 | C15H24O | 73.4 | Terpenoids | C=C1CCC2OC2(C)CCC2C1CC2(C)C | YES |
| Tetrahydropalmatine | 356.1852122 | 4.393533333 | C21H25NO4 | 74 | Alkaloids and derivatives | COC1=C(OC)C=C2C(=C1)CCN1CC3=C(C=CC(OC)=C3OC)CC21 | YES |
| Uridine | 243.0622764 | 0.948616667 | C9H12N2O6 | 73.5 | Nucleotides and derivatives | O=C1C=CN(C2OC(CO)C(O)C2O)C(=O)N1 | NO |
| Benzoic aldehyde | 107.0493885 | 2.325183333 | C7H6O | 76 | Others | O=CC1=CC=CC=C1 | YES |
| Wedelolactone | 313.0358442 | 6.029283333 | C16H10O7 | 70.2 | Flavonoids | COC1=CC(O)=C2C(=C1)OC(=O)C1=C2OC2=CC(O)=C(O)C=C21 | YES |
| Phloridzin | 435.1303742 | 4.91825 | C21H24O10 | 70.1 | Flavonoids | O=C(CCC1=CC=C(O)C=C1)C1=C(O)C=C(O)C=C1OC1OC(CO)C(O)C(O)C1O | NO |
| Esculetin | 179.0338952 | 1.74585 | C9H6O4 | 65.4 | Coumarins and derivatives | O=C1C=CC2=CC(O)=C(O)C=C2O1 | YES |
| Corydaline | 370.2008481 | 5.170233333 | C22H27NO4 | 65.6 | Alkaloids and derivatives | COC1=C(OC)C=C2C(=C1)CCN1CC3=C(C=CC(OC)=C3OC)C(C)C21 | YES |
| Apigenin 7-O-glucoside | 431.0989575 | 4.578333333 | C21H20O10 | 67 | Flavonoids | O=C1C=C(C2=CC=C(O)C=C2)OC2=CC(OC3OC(CO)C(O)C(O)C3O)=CC(O)=C12 | NO |
| Trigonelline | 138.0549463 | 0.61085 | C7H7NO2 | 59.9 | Alkaloids and derivatives | C[N+]1=CC=CC(C(=O)[O-])=C1 | YES |
| Luteolin | 285.0408838 | 5.756066667 | C15H10O6 | 62.7 | Flavonoids | O=C1C=C(C2=CC(O)=C(O)C=C2)OC2=CC(O)=CC(O)=C12 | YES |
| 4-Hydroxy-3-methoxycinnamaldehyde | 177.0551732 | 4.714883333 | C10H10O3 | 63.9 | Others | COC1=C(O)C=CC(C=CC=O)=C1 | YES |
| Emodin | 271.0599008 | 4.28725 | C15H10O5 | 73.8 | Quinones | CC1=CC2=C(C(=O)C3=C(C=C(O)C=C3O)C2=O)C(O)=C1 | YES |
| Quercitrin | 447.0940656 | 4.441233333 | C21H20O11 | 62.1 | Flavonoids | CC1OC(OC2=C(C3=CC(O)=C(O)C=C3)OC3=CC(O)=CC(O)=C3C2=O)C(O)C(O)C1O | NO |
| Vanillic acid | 213.0402317 | 2.45115 | C8H8O4 | 59.3 | Phenolic acids and derivatives | COC1=C(O)C=CC(C(=O)O)=C1 | YES |
| Scopoletin | 193.0496556 | 3.567016667 | C10H8O4 | 56.9 | Coumarins and derivatives | COC1=C(O)C=C2OC(=O)C=CC2=C1 | YES |
| 5,7-dihydroxy-4-methylcoumarin | 237.040542 | 4.509783333 | C10H8O4 | 53.4 | - | CC1=CC(=O)OC2=CC(O)=CC(O)=C12 | YES |
| Azelaic acid | 187.0971497 | 4.827233333 | C9H16O4 | 51.1 | Lipids | O=C(O)CCCCCCCC(=O)O | YES |
| 6-Shogaol | 277.1797478 | 12.69265 | C17H24O3 | 47.1 | Others | CCCCCC=CC(=O)CCC1=CC(OC)=C(O)C=C1 | YES |
| L-Tyrosine | 164.0705297 | 1.082133333 | C9H11NO3 | 52.6 | Amino acids and derivatives | NC(CC1=CC=C(O)C=C1)C(=O)O | YES |
| Dichotomitin | 715.1315106 | 4.75955 | C18H14O8 | 60 | Flavonoids | COC1=CC(C2=COC3=CC4=C(OCO4)C(O)=C3C2=O)=CC(O)=C1OC | YES |
| Sucrose | 341.109212 | 0.607 | C12H22O11 | 44.4 | Carbohydrates and derivatives | OCC1OC(OC2(CO)OC(CO)C(O)C2O)C(O)C(O)C1O | NO |
| Fraxetin | 241.0705438 | 4.67295 | C10H8O5 | 43.5 | Coumarins and derivatives | COC1=C(O)C(O)=C2OC(=O)C=CC2=C1 | YES |
| Dulcitol | 181.0712028 | 0.565666667 | C6H14O6 | 43.3 | Carbohydrates and derivatives | OCC(O)C(O)C(O)C(O)CO | NO |
| Naringenin | 253.0508885 | 5.394416667 | C15H12O5 | 38.8 | Flavonoids | O=C1CC(C2=CC=C(O)C=C2)OC2=CC(O)=CC(O)=C12 | YES |

## 3. Supporting figures


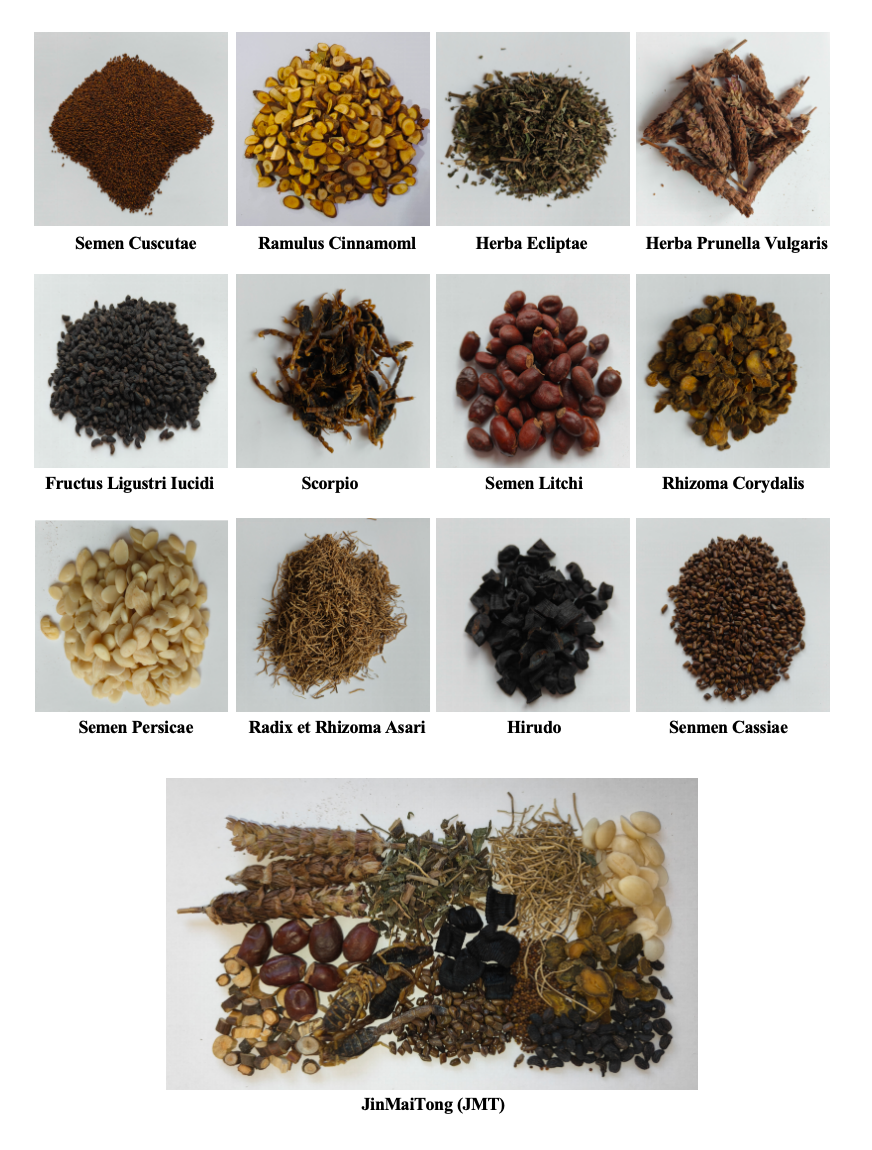


Fig. S1. Twelve kinds of crude drugs in Jinmaitong (JMT) decoction.


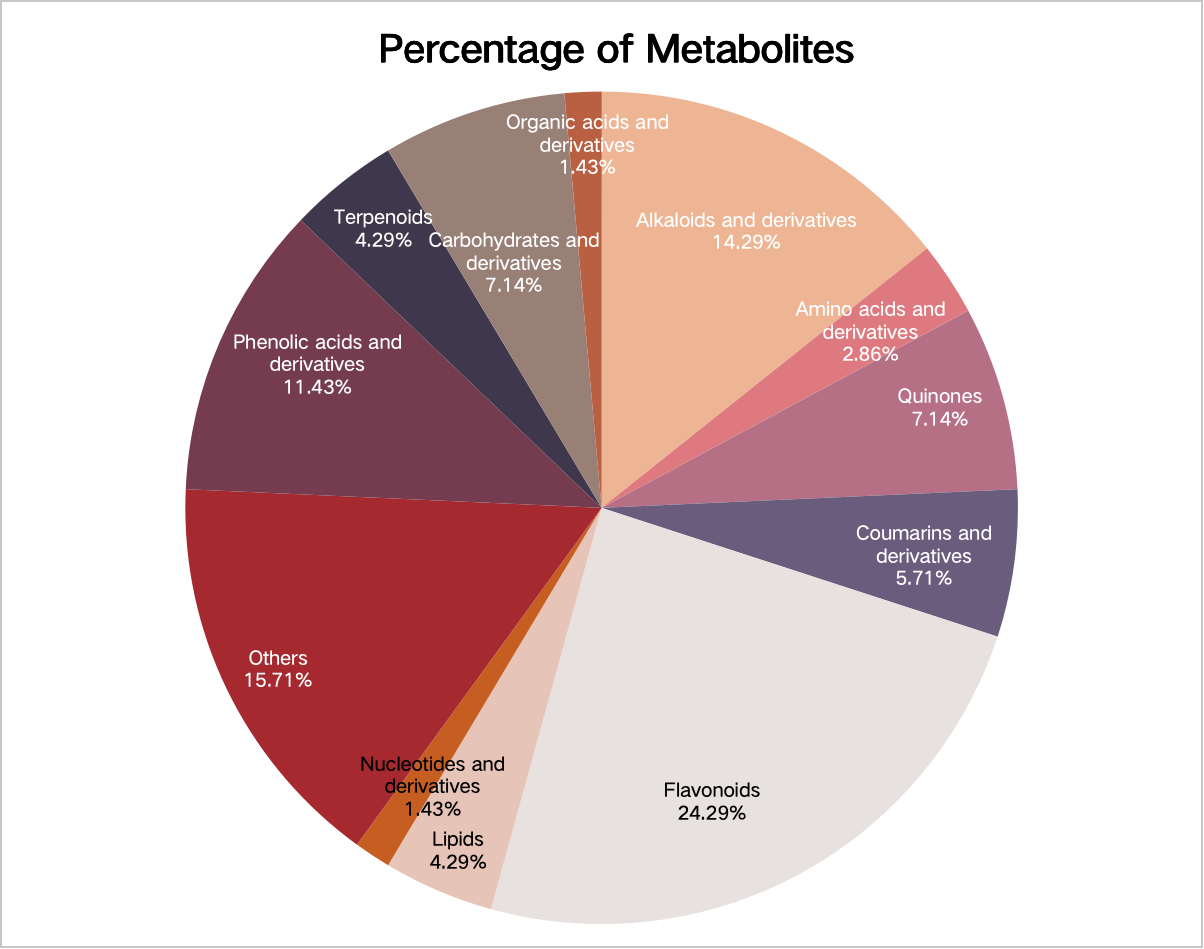


Fig. S2. Proportional distribution of metabolite classes in Jinmaitong (JMT).


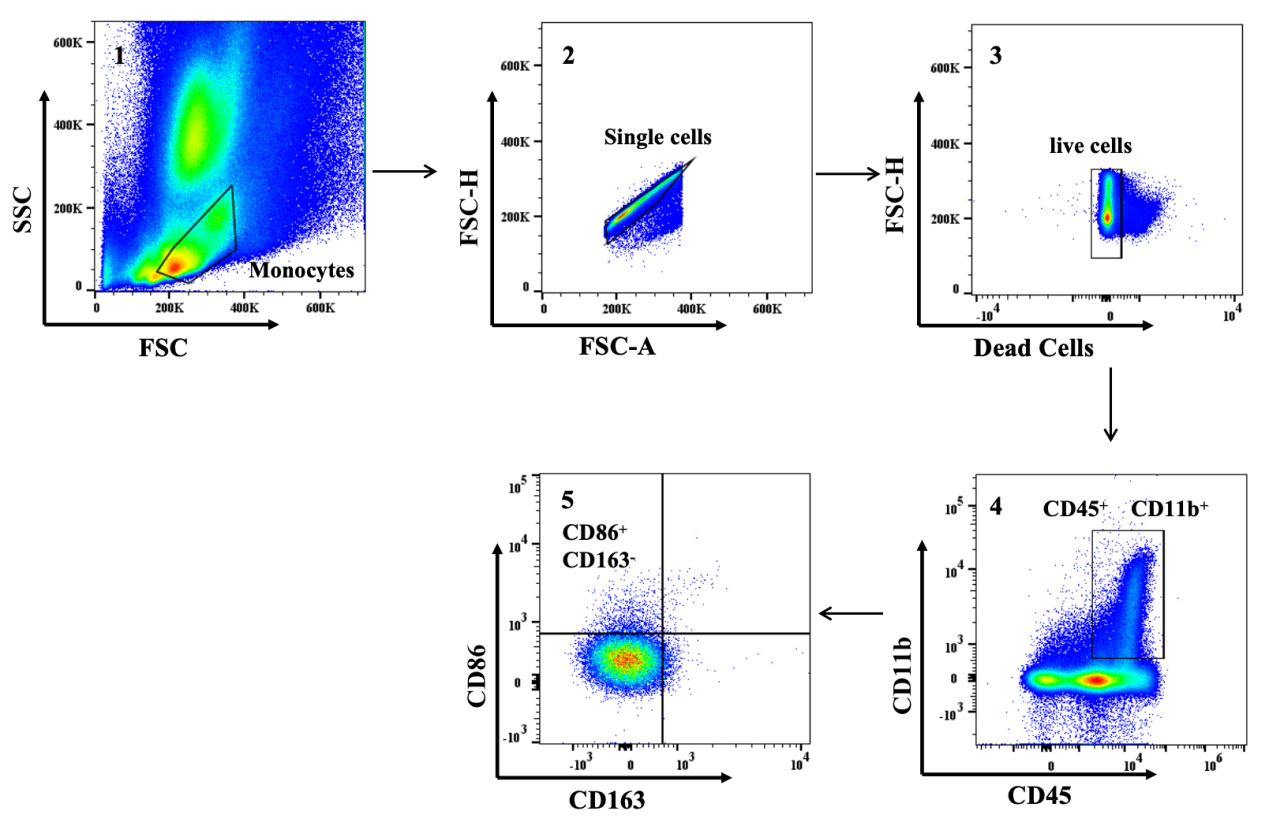


Fig. S3. Gating strategy diagram for flow cytometry.
